# Neural Correlates of Listening to Varying Synchrony Between Beats in Samba Percussion and Relations to Feeling the Groove

**DOI:** 10.3389/fnins.2022.779964

**Published:** 2022-02-25

**Authors:** Annerose Engel, Sebastian Hoefle, Marina Carneiro Monteiro, Jorge Moll, Peter E. Keller

**Affiliations:** ^1^Cognitive and Behavioral Neuroscience Unit, D’Or Institute for Research and Education, Rio de Janeiro, Brazil; ^2^Max Planck Institute for Human Cognitive and Brain Sciences, Leipzig, Germany; ^3^Clinic for Cognitive Neurology, University Hospital Leipzig, Leipzig, Germany; ^4^The MARCS Institute for Brain, Behaviour and Development, Western Sydney University, Penrith, NSW, Australia; ^5^Department of Clinical Medicine, Center for Music in the Brain, Aarhus University, Aarhus, Denmark

**Keywords:** groove, synchrony, human fMRI, rhythm perception, supplementary motor area, subgenual cingulate cortex, music

## Abstract

Listening to samba percussion often elicits feelings of pleasure and the desire to move with the beat—an experience sometimes referred to as “feeling the groove”- as well as social connectedness. Here we investigated the effects of performance timing in a Brazilian samba percussion ensemble on listeners’ experienced pleasantness and the desire to move/dance in a behavioral experiment, as well as on neural processing as assessed via functional magnetic resonance imaging (fMRI). Participants listened to different excerpts of samba percussion produced by multiple instruments that either were “in sync”, with no additional asynchrony between instrumental parts other than what is usual in naturalistic recordings, or were presented “out of sync” by delaying the snare drums (by 28, 55, or 83 ms). Results of the behavioral experiment showed increasing pleasantness and desire to move/dance with increasing synchrony between instruments. Analysis of hemodynamic responses revealed stronger bilateral brain activity in the supplementary motor area, the left premotor area, and the left middle frontal gyrus with increasing synchrony between instruments. Listening to “in sync” percussion thus strengthens audio-motor interactions by recruiting motor-related brain areas involved in rhythm processing and beat perception to a higher degree. Such motor related activity may form the basis for “feeling the groove” and the associated desire to move to music. Furthermore, in an exploratory analysis we found that participants who reported stronger emotional responses to samba percussion in everyday life showed higher activity in the subgenual cingulate cortex, an area involved in prosocial emotions, social group identification and social bonding.

## Introduction

Imagine yourself among a hundred head strong percussion section of a samba school during a carnival parade in Brazil: you and the people around you are singing, dancing, and ecstatically happy, and you have goose bumps all over while feeling a close connection with the people around you. What causes such strong emotional engagement, feelings of pleasure, and an irresistible urge to move along when listening to beating drums? The desire to move and dance with music, especially with highly rhythmic music, together with the experience of feeling pleasure has been described as “feeling the groove” (Madison, 2006; Madison et al., 2011; Janata et al., 2012). Furthermore, such strong emotional experiences in human groups have been observed in the context of religious rituals, where Emile Durkheim referred to it in 1912 as the phenomenon of “collective effervescence”—“a kind of electricity that quickly transports them [individuals in a group] to an extraordinary degree of exaltation” (Durkheim, 2008, p. 162). Even in the laboratory, experiencing pleasurable music can lead to peak emotional responses marked by “chills” that are correlated to physiological arousal (Panksepp, 1995; Grewe et al., 2007, 2009; Salimpoor et al., 2009; Witek, 2009).

### Factors That Influence “Feeling the Groove”

There is a growing body of studies examining the underpinnings of “feeling the groove,” namely the physical properties of music that correlate with this unique experience and potentially cause or contribute to it. Predictive coding accounts have proposed that musical expectations, which are continuously formed and then fulfilled or violated to varying degrees during music listening, mediate the pleasurable experience of groove-based music (Vuust and Witek, 2014; Vuust et al., 2018; Koelsch et al., 2019). Studies of groove induction have consistently highlighted the impact of rhythm-related factors that increase the amount of temporal information and influence musical expectations. These factors include beat salience (i.e., the degree to which the perception of a periodic beat is encouraged by the rhythmic patterning of sound events), the relative magnitude of periodic sound events at metrical levels faster than the beat, the density of sound events between beats, and higher-order characteristics of rhythms like syncopation (a shift of rhythmic emphasis from metrically strong to metrically weak beats; Madison et al., 2011; Madison and Sioros, 2014; Witek et al., 2014). Some of these rhythmic factors (e.g., beat salience) have a linear relationship to feeling groove (e.g., higher groove ratings in music are related to increasing beat salience and the density of sound events between beats; Madison et al., 2011). By contrast, higher order factors such as syncopation have been found to have an inverted U-shaped relation, with highest groove ratings for medium levels of rhythmic complexity (Witek et al., 2014; Matthews et al., 2019, 2020).

Furthermore, studies of the effects of microtiming on the experience of groove have revealed mixed results (Madison and Sioros, 2014; Senn et al., 2017). “Microtiming” refers to small temporal deviations from beats (defined relative to theoretical timepoints associated with metrical structure) that occur at a millisecond timescale. These deviations increase temporal uncertainty but also play an important role in communicating expressive aspects of music performance (Keil, 1995; Palmer, 1997; Keller et al., 2014). Keil proposed in his theory of “participatory discrepancies” (Keil, 1995) that the tension created by asynchronous timing between bass and drum is important for creating groove music. This view is widely shared among musicians (Berliner, 1994), but experimental studies aimed at testing it have yielded contradictory results (Senn et al., 2017; Datseris et al., 2019).

Madison et al. (2011) differentiated between systematic (repetitive or intentional) microtiming, which is related to intended expressive shifts in music performance, and non-systematic (non-repetitive or unintentional) microtiming, related to human limits in perception and motor control. In their study of different musical genres (Greek, Indian, Jazz, Brazilian samba, and West African music), no relation was found between unsystematic microtiming and groove, but correlations with systematic microtiming were positive in Brazilian samba (larger microtiming deviations were related to higher groove ratings) and negative in Greek music (greater isochrony was associated with increased groove ratings).

While this study and others focused on the magnitude of microtiming deviations, Senn et al. (2017) studied the patterning of microtiming deviations by comparing fixed time shifts in naturalistic recordings of a drum and bass duo, playing swing or funk. Their results indicated that phase shifts between instrumental parts (displacing e.g., the entire drum track relative to the bass track by certain amounts) did not influence listeners’ groove experience but shifts within instrumental parts (only displacing the snare drum, while keeping the bass track and other drum parts in their original temporal position) had a negative effect on groove experience. Furthermore, a comparison of the effects of such fixed microtiming displacements with locally applied manipulations, using scaled versions of originally performed microtiming patterns in naturalistic recordings, revealed that fixed snare drum displacements irritated expert listeners more than the flexible deviations occurring in the original performances. Together these findings suggest that the effects of microtiming deviations on the experience of groove are manifold and depend on the type of timing deviation.

Finally, beat tempo has been found to be only weakly related to groove (Madison et al., 2011), though listeners’ experiences of groove are strongest at a range of tempi corresponding to preferred movement rates (Etani et al., 2018). Madison et al. (2011) explored several factors in their study of different musical genres, including Brazilian samba, and found evidence for universal effects of groove across different styles. However, it is noteworthy that Brazilian samba was found to have higher potential than other genres to induce groove. This result corroborates our choice for selecting samba rhythms for inducing groove and associated emotional experiences.

### Neural Basis of “Feeling the Groove” and Rhythm Processing

To identify the neural correlates of groove, a recent functional magnetic resonance imaging (fMRI) study investigated the feeling of groove while listening by manipulating rhythmic and harmonic complexity in musical stimuli (rhythmic piano chord patterns, Matthews et al., 2020). This study confirmed that the sensation of groove is related to processing in motor and reward networks in the brain. The authors proposed a theoretical model to account for how the interaction of brain areas within different cortico-striatal circuits supports internal representations of the beat (putamen, supplementary motor area and premotor area) and beat-based musical expectations (caudate, prefrontal and parietal regions). Both circuits pass information to a reward network (nucleus accumbens and medial orbitofrontal cortex) that generates the typical response to groove, i.e., the feeling of pleasure and the desire to move.

Relatedly, the formation of musical expectations is central to “predictive coding” theoretical frameworks of music listening (Vuust and Witek, 2014; Vuust et al., 2018; Koelsch et al., 2019). More generally, predictive coding is considered to be fundamental for action and perception, describing the process of generating and updating internal representations of the environment by comparing predicted (top–down) with actual sensory input (bottom–up) using prediction errors for updating. The generation of internal representations during perception has also been linked to action-simulation processes involving the motor system (Hommel et al., 2001; Rizzolatti and Craighero, 2004; Schubotz, 2007). In line with this, the sensory-motor theory of rhythm perception (Todd and Lee, 2015) claims that rhythm and beat experience involve a sensory representation of the input as well as a motor representation of the body. In support of this theory, rhythm processing and beat perception are associated with activation of motor areas in the brain, including the supplementary motor area, premotor cortex, basal ganglia, and cerebellum (Chen et al., 2008; Bengtsson et al., 2009; Grahn and Rowe, 2009; Kornysheva et al., 2010; Danielsen et al., 2014). Moreover, listening to musical rhythms that are judged to be beautiful (or liked) leads to higher activation in premotor and cerebellar areas than listening to non-preferred rhythms (Kornysheva et al., 2010), and musical groove modulates the motor cortex excitability in musicians (Stupacher et al., 2013).

### Sensorimotor Synchronization, Rhythmic Entrainment, and Emotional Processing

Another line of research that motivates the present study addresses sensorimotor synchronization and rhythmic entrainment. With respect to functional relevance, the experience of groove in music is associated with better synchronization and effortless coordination of movements due to optimal sensorimotor coupling (Merker et al., 2009; Janata et al., 2012; Leow et al., 2014, 2021; Fitch, 2016; Witek et al., 2017). Furthermore, interpersonal synchrony during joint musical activities has become a topic of intense interest because of its positive effects on cooperation, prosocial behavior, and interpersonal affiliation. For example, joint drumming or finger tapping in synchrony increases subsequent cooperation between the co-acting individuals (Kirschner and Tomasello, 2009; Kokal et al., 2011), feelings of affiliation (Hove and Risen, 2009), and activation of brain areas involved in reward processing (Kokal et al., 2011). Kokal et al. (2011) showed stronger activation in the caudate, an intersection between reward and motor networks in the brain, when participants experienced higher interpersonal synchrony during dyadic drumming. This activation was not only related to reward, but also predicted the amount of prosocial behavior engaged in afterward, and was furthermore mediated by the interindividual differences in the ease with which participants learned to produce the joint rhythm.

As mentioned earlier, it is assumed that action and perception share common neural substrates (Hommel et al., 2001; Rizzolatti and Craighero, 2004). Consistent with this general principle, and its specific instantiation in the sensory-motor theory of rhythm perception (Todd and Lee, 2015), it is assumed that beat perception entails a covert action simulation process that involves triggering motor representations that would be necessary for action execution and for predicting action outcomes. Relatedly, dynamic attending theory (Large and Jones, 1999) proposes that temporal prediction emerges via process of entrainment, a phenomenon whereby two or more “systems” become coupled. This coupling can take place at several levels in the context of music (neural, perceptual, autonomic physiological, motor, social, subjectively; see Trost et al., 2017). Notably, there is evidence that emotional experience is related to entrainment during listening to percussion (Trost et al., 2014, 2017; Cameron et al., 2019).

Thus, the perception of an action such as listening to samba percussion, which requires high interpersonal synchronization (i.e., good synchrony among instruments or playing “in sync”), can be expected to recruit similar brain areas as executing that action, including motor regions and reward regions, and brain areas related to prosocial behavior and emotional processing, in particular feeling connected with others. Furthermore, these processes can be expected to be facilitated by high synchrony between percussion instruments due to enhanced action simulation and entrainment at multiple levels leading to stronger “resonance” in the system.

With regard to emotional processing during music listening, Becker (2004) proposed that some individuals show profound emotional experiences when listening to music. Such “deep listeners” show enhanced physiological responses (e.g., goosebumps, a racing heartbeat) to music and describe themselves as having relatively pronounced emotional responses when listening to music (Becker, 2004). Becker noted the relationship between such pronounced emotional experiences during music listening and experiences of trance and ecstasy in religious ceremonies (cf., Durkheim, 2008). However, only a handful of studies have examined deep listeners. For example, Penman and Becker (2009) showed that deep listeners have higher galvanic skin responses, indicating stronger emotional responses, than individuals from various control groups, and Chapin et al. (2010) employed deep listeners to investigate the neural correlates of emotional responses during natural music listening. Such depth of emotional responses to music on a trait level might also contribute to the above-mentioned prosocial behavior and affiliative emotions when experiencing synchrony in musical ensembles. The subgenual cingulate cortex is an area that has been implicated in attachment, affiliative emotions, altruistic decisions, prosocial behavior, emotional processing, and ingroup-related effort (Bartels and Zeki, 2004; Aron et al., 2005; Moll et al., 2006, 2012; Krueger et al., 2007; Zahn et al., 2009; Rusch et al., 2014; Bortolini et al., 2017) and thus might be the intersection for emotional experience that facilitates affiliative emotions and prosocial behavior. In a relevant study, Rusch et al. (2014) investigated the degree to which participants perceive their family as a distinct and cohesive group (high entitativity). They found, in concordance with the role of the subgenual cingulate cortex in affiliation (Moll et al., 2012), that increased activity in the subgenual cingulate cortex was related to high entitativity reflecting group belongingness. The authors conclude that the subgenual cingulate cortex may represent the link between kin-related emotional attachment and group perception.

### Outline of the Present Study

In the present study we aimed to bring together different lines of research on experiencing synchronous action and feeling the groove in rhythmic musical sounds in order to explore links between synchrony perception, in particular its temporal properties, and affective experiences induced by music. To this end, we investigate the effect of varying degrees of synchrony between multiple instrumental parts from a samba percussion ensemble on experienced pleasantness and neural processing during listening. More specifically, we used naturalistic sounds of high-quality professional recordings of a samba percussion ensemble, namely the rhythm or percussion section (*Bateria*) of a Brazilian samba school (*Escola de samba*^1^). These recordings consisted of sounds played “in sync” (with natural microtiming deviations occurring in the naturalistic recordings but with no additional asynchrony between instrumental parts) or manipulated sounds played “out of sync” (with varying degrees of asynchrony added to the naturalistic recordings, creating fixed time shifts between instrumental parts). For simplicity, the sounds of the percussion section of a Brazilian samba school will be referred to as “samba percussion” throughout the manuscript.

First in a behavioral study, we investigated how: (1) varying degrees of synchrony between multiple instrumental parts; and (2) loudness influence the pleasurable experience of a listener and the desire to move/dance with the samba percussion sounds. We hypothesized that listening to more synchronous stimuli would induce: (a) greater pleasantness; and (b) stronger desire to move/dance. This is motivated by the assumption that: (a) experiencing synchrony (compared to experiencing asynchrony) between instruments during joint drumming triggers activity in brain areas processing reward (and might be related to the rhythmic abilities of drummers, Kokal et al., 2011); and (b) rhythm processing and beat perception are related to activation of motor areas in the human brain (e.g., Chen et al., 2008; Grahn and Rowe, 2009) that facilitate the desire to move/dance.

Furthermore, we assumed that very loud stimuli would induce greater pleasantness and a stronger desire to move/dance than less loud versions of the stimuli. An extension of the sensory-motor theory of rhythm perception (Todd and Lee, 2015) proposes that rhythm perception is a form of vestibular perception by indicating overlapping brain areas in vestibular and rhythm processing. In addition, the authors assume that the neuroanatomical and functional connections of the vestibular system with cortico-subcortical regions involved in emotion and motivation (Rajagopalan et al., 2017) are the basis of a reward-based learning mechanism that underlies the compulsion to move with a beat. The involvement of the vestibular system in rhythm perception has further implications. Todd and Cody (2000) have shown that samples of loud techno music (above 90 dB sound pressure level, SPL) elicited a greater vestibular response and suggest that vibrotactile stimulation might be the source of pleasure, as such stimulation is sought in self-selected motion as in swings, rocking chairs or fun parks, and even in a self-evoked motion like head banging to music. Thus, vestibular responses may account for pleasurable sensations during listening to loud rock and dance music. With regard to our stimuli, we expected an effect of loudness only when the instruments in the percussion section are “in sync”. We assume that a higher beat salience is conveyed by “in sync” stimuli (due to unambiguous timing cues) especially when presented very loud, in which case there will be a stronger vestibular response. In contrast, sounds played “out of sync” e.g., sounds with varying degrees of asynchrony between instrumental parts, have rhythmical properties that would be more difficult for actions simulation processes during perception and this would also influence the vestibular response and relations to reward. Finally, we examined (3) whether inter-individual differences in rhythm and time perception abilities are related to the experience of pleasantness and the processing of asynchronies in samba percussion.

Second, in a fMRI study, we investigated the effects of varying degrees of synchrony between multiple instrumental parts on experienced pleasantness and its neural processing while amateur musicians listened to samba percussion. We also assessed the degree to which participants were “deep listeners” (Becker, 2004; Chapin et al., 2010), based on self-reports of emotional responsiveness to samba percussion in daily life, to explore the potential relevance of this construct to inter-individual differences in neural processing. Based on evidence that emotional experiences are related to neural entrainment during music listening (Trost et al., 2014, 2017; Cameron et al., 2019), we expected that such experiences would be enhanced by higher synchrony between samba percussion instruments. We selected the subgenual cingulate cortex as an *a priori* region of interest for additional analyses because of its involvement in emotional processing, including attachment and affiliative emotions related to prosocial behavior and social group identification (Bartels and Zeki, 2004; Aron et al., 2005; Moll et al., 2006, 2012; Krueger et al., 2007; Zahn et al., 2009; Rusch et al., 2014; Bortolini et al., 2017). We were specifically interested in whether differential responses in this region of interest are related to individual differences in the self-reported intensity of emotional responses when listening to samba percussion in daily life.

## Materials and Methods

### Participants

#### Behavioral Study

Twelve volunteers (mean ± standard deviation, SD: 30.6 ± 6.9 years, range: 20–40 years, eight female) participated in the study. Eight of the 12 participants played one or more instrument(s) and all participants reported that they were familiar with and liked listening to the sounds and rhythms of samba percussion (see Supplementary Material 1.1 Participants for information about their musical background).

#### Functional Magnetic Resonance Imaging Study

The final sample for the fMRI experiment comprised 21 new volunteers (mean age ± SD: 34.4 ± 5.5 years; range: 26–42, five female), none of whom had participated in the behavioral study. All of these participants had musical experience (16 amateur and five professional musicians), played an instrument, and were familiar with and liked listening to the sounds and rhythms of samba percussion (see Supplementary Material 1.1 Participants for the detailed information about musical background). 19 out of the 21 participants reported that they were able to play the typical samba percussion rhythm. None of the participants had a history of neurological disorders or were taking centrally active medications. 20 participants were right-handed and one participant was left-handed according to the Edinburgh handedness inventory (Oldfield, 1971).

All participants in both studies had normal vision or corrected to normal vision (contact lenses) and normal hearing abilities. They gave their written informed consent to participate and were naïve to the hypotheses and manipulation of the stimuli. The experiments were performed in accordance with ethical standards compliant with the declaration of Helsinki and had been approved by the local scientific ethics committee (Copa D’Or Hospital, Rio de Janeiro, Brazil, No. 57.482).

### Stimuli

**FIGURE 1:**
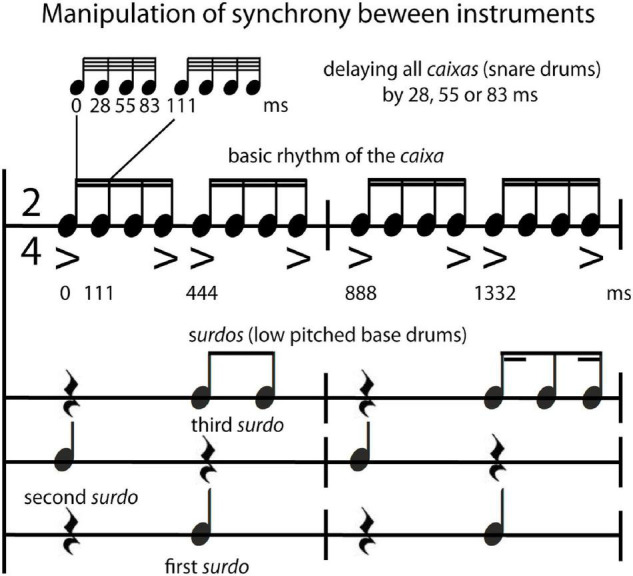
Stimulus manipulation. Notation of the typical rhythm played by the percussion section of a Brazilian samba school (samba percussion) with the experimental manipulation that entailed shifting the *caixas* (snare drums) by 0, 28, 55, 83 ms in relation to the *surdos* (low pitched bass drums).

The typical sequence performed by a Brazilian samba school percussion section was arranged and recorded by a professional musician in Rio de Janeiro using an overdubbing procedure on multiple audio tracks (tempo: 135 bpm; 2/4 bar; 135 s; including two parts each with a refrain = *bossa*; see Supplementary Material, 1.2 Recording of stimuli for a comprehensive description and analyses of stimulus features). The following instruments were included in this multitracked percussion section: *repinique*—high pitch double-headed drum; *surdos*—low pitched bass drums (three different versions, the first, second and third *surdo*), *caixas*—snare drums, *chocalhos*—shakers, *cuícas*—high-pitched Brazilian friction drums, *tamborins*—small frame drums, and *agogôs*—agogo bells. For the behavioral study, an excerpt from the beginning of the recording (24 s) was used, consisting of the call of the *repinique* (4 bars long), the entrance of the *surdos*, *caixas and tamborins*, and, finally, 14 bars after the start, the entrance of *chocalhos, cuícas*, and *agogôs*. In order to increase the number of trials for the fMRI experiment, three different excerpts (each 19.555 s, either containing the call of *repinique* or a break/refrain—*bossa*) of the recording were used. The synchrony manipulation was applied to these excerpts by using Logic Pro 9 (Apple Inc., Cupertino, CA, United States) either to align the tracks of the single instruments “in time/in sync” (0 ms delay between instrumental parts) or to delay the *caixas* (snare drums that play the underlying beat of the rhythm) by 28 ms, 55 ms or 83 ms (“out of time/out of sync”). These time shifts are multiples of beat subdivisions at the 64th-note level (see Figure 1 for relations of the rhythms and accents between the *caixas*—and the three different *surdos;* see also Supplementary Material 3 Listening Examples Audio 1–4). Note that stimuli that were used to create the time shifts for the synchrony manipulation comprise naturalistic recordings that contain microtiming deviations that occur normally in live performances. Furthermore, sound intensity of stimuli used in the behavioral study was manipulated by normalizing the mean root mean square (RMS) of the audio waveform, resulting in very loud (95 dB SPL) and loud (85 dB SPL) versions of stimuli. There was no manipulation of sound intensity for the stimuli in the fMRI study (all stimuli were presented with the same loudness).

### Experimental Procedure and Data Analyses for the Behavioral Study

In the behavioral study, participants were tested while seated in an experimental room. Stimulus delivery was controlled by Presentation 16.4 software (Neurobehavioral Systems Inc., Berkely, CA, United States) running on a computer and stimuli were heard via Sennheiser HD280PRO headphones (Sennheiser Electronic GmbH & Co. KG, Wedemark, Germany). The experimental procedure in the behavioral study comprised: (1) perception tasks that assessed, first, the experienced pleasantness and desire to move/dance and, second, the ability to perceive timing shifts in percussion sounds, both using the stimuli described above; (2) tests of participants’ general rhythmic and auditory perceptual abilities; and (3) questionnaires.

(1) The experimental design for the first perception task was based on a 2 (task: pleasantness/desire to move rating) × 2 (loudness: very loud/loud stimuli) × 4 (synchrony between instrumental parts: 0, 28, 55, or 83 ms delay of snare drums) factorial design. In each trial, participants were required to listen to a stimulus sequence and to judge their experienced pleasantness and their desire to move/dance with it (both on a rating scale from 0 = “not at all” to 10 = “very much”). All stimuli were presented once in a randomized order. In the second perception task, participants’ ability to detect asynchronies between percussion parts was examined for the four different levels of synchrony (0, 28, 55, or 83 ms delay of snare drums) only for the very loud stimuli (95 dB SPL). In each trial, participants were required to judge the degree to which the instruments were being played in time (i.e., in synchrony) on a rating scale ranging from 0 = “not at all synchronously” to 10 = “perfectly synchronized”). This test was repeated (i.e., done twice), and all stimuli were presented in a randomized order. For calculating an index of the sensitivity to the timing shifts in percussion sounds, each participant’s (subjective) ratings for perceived synchrony between instruments were correlated with the (objective) values (0, 28, 55, 83) used to delay the snare drums using the Pearson correlation coefficient *r*.

(2) In order to evaluate participants’ general rhythmic abilities, the rhythmic part of the Musical Ear Test (MET; Wallentin et al., 2010) was used. Participants were required to compare two rhythm patterns by judging whether the patterns were same or different. Presentation of each pair of rhythm patterns started with 4 metronome clicks (100 bpm, 4/4 bar) followed by a sequence of 4–11 wood block sounds (one bar, first rhythmical phrase), further metronome clicks to complete the second bar, a sequence of 4–11 wood block sounds (one bar, second rhythmical phase) and further metronome clicks to complete the fourth bar. Fifty-two pairs of rhythm patterns that varied in difficulty were presented, with half of the pairs containing change in the second pattern.

In order to evaluate participants’ perceptual abilities, an adaptation of an auditory flutter fusion task (Rammsayer and Altenmueller, 2006) was created to determine perceptual thresholds for perceiving two sounds as being simultaneous. Stimuli consisted of sound pairs created by two bongo sounds (one with a high pitch, the other with a low pitch) that were separated by a gap ranging from 0 to 200 ms. In each experimental trial, two synchronous (0 ms gap) and one sound pair that contained a gap (deviant stimulus) were presented. Participants indicated which of the three sound pairs had a gap. Stimuli presentation followed an adaptive staircase procedure (see Janata and Paroo, 2006): if the participant responded correctly, the gap in the deviant stimulus decreased in the following trial, whereas the gap increased for the next trial in following incorrect responses. The adaptive testing procedure required participants to have ten turnaround points (incorrect answers), and the gap size values for the last correct response before the incorrect response for the last six turnaround points were averaged to determine the perceptual threshold.

(3) Finally, participants completed questionnaires that assessed: (a) their musical background by custom-created questions specific to samba percussion, practicing hours, and experience playing in musical ensembles (see Supplementary Material 1.1 Participants); (b) emotional experience (Geneva Emotional Music Scale, GEMS-9, Zentner et al., 2008) and (c) physiological sensations (based on questionnaires for identifying “deep listeners”, cf. Becker, 2004; Chapin et al., 2010) when listening to the samba percussion in daily life.

Emotional experience in response to samba percussion (in daily life) was assessed by the Geneva Emotional Music Scale (GEMS-9, Zentner et al., 2008). Participants rated the intensity of several musical emotions that they normally feel when listening to samba percussion using a 5-point rating scale (1 = “not at all” to 5 = “very much”): wonder (filled with wonder, dazzled, moved), transcendence (fascinated, overwhelmed, feelings of transcendence and spirituality), power (strong, triumphant, energetic), tenderness (tender, affectionate, in love), nostalgia (nostalgic, dreamy, melancholic), peacefulness (serene, calm, soothed), joyful activation (joyful, amused, bouncy), sadness (sad, sorrowful), and tension (tense, agitated, nervous).

Physiological sensations in response to samba percussion (in daily life) were assessed based on questionnaires designed to identify “deep listeners” (cf. Becker, 2004; Chapin et al., 2010). Participants were required to rate on a 5-point scale (1 = “never” to 5 = “always”) the degree to which they experience physiological sensations when normally listening to samba percussion: goose bumps, sensations in the stomach, tingles down the spine, shivering, heart palpitations, lumps in the throat, a racing heart, synesthesia.

The data analyses were performed with SPSS 20.0 (IBM Corp., Armonk, NY, United States) using multivariate tests for repeated measures Analysis of Variance (ANOVA), paired *t*-tests for comparisons of stimuli, and the Pearson correlation coefficient *r*. Consistent with our directional hypotheses, one-tailed significance testing was applied.

### Experimental Procedure for the Functional Magnetic Resonance Imaging Study

During the fMRI experiment, participants lay in supine position on the scanner bed, with the right hand resting on the scanner-compatible response box. Written instructions were projected by an LCD projector onto a screen behind the participant’s head (viewed via a mirror on the top of the head coil). All auditory stimuli were presented via scanner-compatible headphones (MRconfon GmbH, Magdeburg, Germany). Stimulus delivery was controlled by Presentation 16.4 software (Neurobehavioral Systems Inc., Berkely, CA, United States) running on a computer.

The fMRI experiment comprised 48 experimental trials (12 for each of the four experimental conditions—varying degrees of synchrony of 0, 28, 55, and 83 ms delay) and six null events (20 s silence) that were presented in 2 runs (each 15 min, each comprising 24 experimental trials and three null events). Experimental trials followed the same structure: a fixation cross was presented for 5 s and then additional to that percussion sounds (19.555 s) were presented. Participants were instructed to listen to the percussion stimuli carefully and passively without any movement (see also Supplementary Material 1.3 Control for movement during the fMRI experiment). After listening they were required to rate how pleasant (enjoyable) was listening to the excerpt on a 10-point rating scale with the anchor points 1 = “very unpleasant” and 10 = “very pleasant” that was presented for 10 s (by moving a red circle to the position on the scale). Stimuli with varying degrees of synchrony between instruments were presented across experimental trials in a pseudo-randomized order (see Supplementary Material 1.4 Stimulus presentation order in the fMRI experiment). Participants were familiarized with the task prior to the scanning session using different excerpts than during fMRI scanning. Note that ratings of the desire to move/dance were not collected in the fMRI experiment.

After fMRI scanning, a debriefing session took place on strategies and other aspects of task performance during the fMRI study (see Supplementary Material 1.5 Debriefing after fMRI scanning). As in the behavioral study, participants also completed questionnaires that assessed: (a) their musical background by customized questions specific to samba percussion, practice, and ensemble experience (see Supplementary Material 1.1 Participants); (b) emotional experience (GEMS-9, Zentner et al., 2008) and (c) physiological sensations when listening to the samba percussion in daily life.

Participants were asked to report their emotional experience (using the GEMS-9, Zentner et al., 2008, as in the behavioral study described above) and physiological sensations (according to questionnaires that identify “deep listeners”, cf. Becker, 2004; Chapin et al., 2010) when listening to samba percussion in general. Here (in contrast to the behavioral study) participants rated on a 5-point scale (1 = “never” to 5 = “always”) only five of the most prevalent physiological sensations identified in the behavioral study: goose bumps, sensations in the stomach, tingles down the spine, shivering, a racing heart.

Furthermore, in order to identify “deep listeners”, participants were to judge on a 5-point rating scale whether their emotional responses when listening to samba percussion in daily life are stronger or weaker/equally strong compared to the emotional responses of most people they know (1 = “much less,” 2 = “less,” 3 = “equal,” 4 = “stronger” or 5 = “much stronger”). Participants further indicated the role of samba percussion in their life (1 = “no role at all” to 5 = “great role on their life”) and how strongly they experience emotions when listening to samba percussion (1 = “not at all” to 5 = “intensively”).

Finally, participants’ ability/sensitivity at detecting asynchronies between percussion parts in the stimuli was examined (comparable to the second perception task of the behavioral study). In this behavioral test, participants listened to 24 stimuli (two presentations for each) again outside the fMRI scanner and judged the degree to which the instruments were played in time (i.e., in synchrony) on a rating scale ranging from 1 = “not at all synchronously” to 10 = “perfectly synchronized”. Stimulus delivery was controlled by Presentation 16.4 software (Neurobehavioral Systems Inc., Berkely, CA, United States; running on a computer) and stimuli were heard over Sennheiser HD280PRO headphones (Sennheiser Electronic GmbH & Co., KG, Wedemark, Germany). The presentation order followed constraints described in Supplementary Material 1.4. In order to calculate an index for the sensitivity to the timing shifts in percussion sounds, each participant’s (subjective) ratings for perceived synchrony between instruments were correlated with the (objective) values (0, 28, 55, 83) used to delay the snare drums using the Pearson correlation coefficient *r*, as implemented in Matlab R2012a (The Mathworks Inc., Natick, MA, United States).

Behavioral data analyses were performed with SPSS 20.0 (IBM Corp., Armonk, NY, United States) using multivariate tests for repeated measure Analysis of Variance (ANOVA) and paired *t*-tests for comparisons of stimuli with different degrees of synchrony between instrumental parts.

### MRI Acquisition and Data Analysis

MR scans were performed on a 3 Tesla Philips Achieva MR scanner (Koninklijke Philips N.V., Amsterdam, The Netherlands) with an eight-channel SENSE head coil. Two runs of 360 functional whole brain images, sensitive to the blood oxygenation level dependent (BOLD) signal, were acquired using a single-shot T2*-weighted fast-field echo (FFE) echoplanar imaging (EPI) sequence. Each volume consisted of 39 AC-PC aligned slices covering the whole brain with the following parameters: voxel size 3 mm × 3 mm and a slice thickness of 3.0 mm and 0.75 interslice gap; repetition time (TR) 2.5 s, echo time (TE) 22 ms, flip angle 90°, acquisition matrix 80 × 80, field of view (FOV) 240× 240× 145.5 mm, ascending image acquisition. Before each functional run, five dummy volumes were collected for T1 equilibration purposes. A SENSE factor of 2 and “dynamic stabilization” were additionally employed. These parameters were based on careful sequence parameter optimization in order to maximize temporal signal-to-noise (Bellgowan et al., 2006; Bodurka et al., 2007) in brain regions that normally suffer from magnetic susceptibility effects, including the basal forebrain areas and ventromedial regions of the prefrontal cortex. Additionally, a set of anatomical images was acquired (see Supplementary Material 1.6 fMRI parameter for anatomical images). Head motion was restricted with foam padding and straps over the forehead and under the chin (estimated translation and rotation parameters were inspected and never exceeded 2 mm or 2 degrees).

fMRI data were analyzed using Statistical Parametric Mapping SPM8 implemented in Matlab R2012a (The Mathworks Inc., Natick, MA, United States). All functional images were pre-processed by realigning all volumes of each subject to the first functional volume and in a second step to the mean image. Functional images were co-registered to the 3D anatomical image of the participant. The 3D anatomical image was segmented, and the gray matter segment was normalized to a gray matter template corresponding to the Montreal Neurological Institute (MNI) brain template and obtained parameters were used for normalization of the functional data. The voxel dimensions of each reconstructed functional scan were 3 × 3 × 3 mm. Finally, functional images were spatially smoothed with a 6 mm full-width half-maximum Gaussian filter. In the first-level analysis, pre-processed images of each participant were analyzed with a General Linear Model (GLM) according to a factorial design using the four experimental conditions (varying degrees of synchrony between instruments). For each functional run, the GLM model of the first level included four predictors of interest covering the experimental manipulations, namely the delay between the snare drums (*caixa*) and the other instruments in the samba percussion stimuli: (1) 0 ms; (2) 28 ms; (3) 55 ms; (4) 83 ms and were modeled as boxcar functions with a length of 19.555 s convolved with the canonical hemodynamic response function. Moreover, a predictor (of no interest) that covered the time for presenting the rating scale and the response of participants was included by modeling a boxcar function with a length of 10 s convolved with the canonical hemodynamic response function. The six movement parameters were included as further predictors of no interest. Low frequency drifts from the perceptual functional runs were removed using a high pass filter of 516 s and a correction for autocorrelation [AR(1)] was applied. In the first level analyses, a contrast was generated that tested for increasing responses with increasing synchrony between instruments: 0 > 28 > 55 > 83 ms (i.e., the interaction contrast [2 1 -1 -2]). These contrast images of single participants were used in a second-level one-sample *t*-test for random effects analysis. Additionally, a parametric analysis was conducted with the index that describes the rhythmic abilities of the participants (correlation coefficients obtained from the synchrony judgment task). For identified areas, we first report activations that were significant at *p* < 0.05, corrected for multiple comparisons (using Family Wise Error Rate, FWE, on a cluster level) with a minimum cluster size of 10 voxels. Additionally, we report the results in an exploratory manner at a significance level of *p* < 0.005, uncorrected for multiple comparisons, and activation clusters with minimum size of 10 voxels. Displays of activations were created by means of software packages MRIcron^2^ by superimposing SPM *t*-maps resulting from the second level analysis maps on a MNI standard brain. Labeling of activation clusters was done with the anatomy toolbox^3^, bspmview^4^ and brain atlases. Region of interest analysis was done using MarsBaR (Brett et al., 2002)^5^ by extracting parameter estimates in the contrast of increasing synchrony between instruments (0 > 28 > 55 > 83 ms) at a predefined coordinate for the subgenual cingulate cortex (Moll et al., 2006; x = –2, y = 15, z = –5, using a 10 mm sphere), and for control to an anatomical mask for the nucleus accumbens (Pauli et al., 2018).

## Results

### Behavioral Study

#### Perception Tasks

**FIGURE 2:**
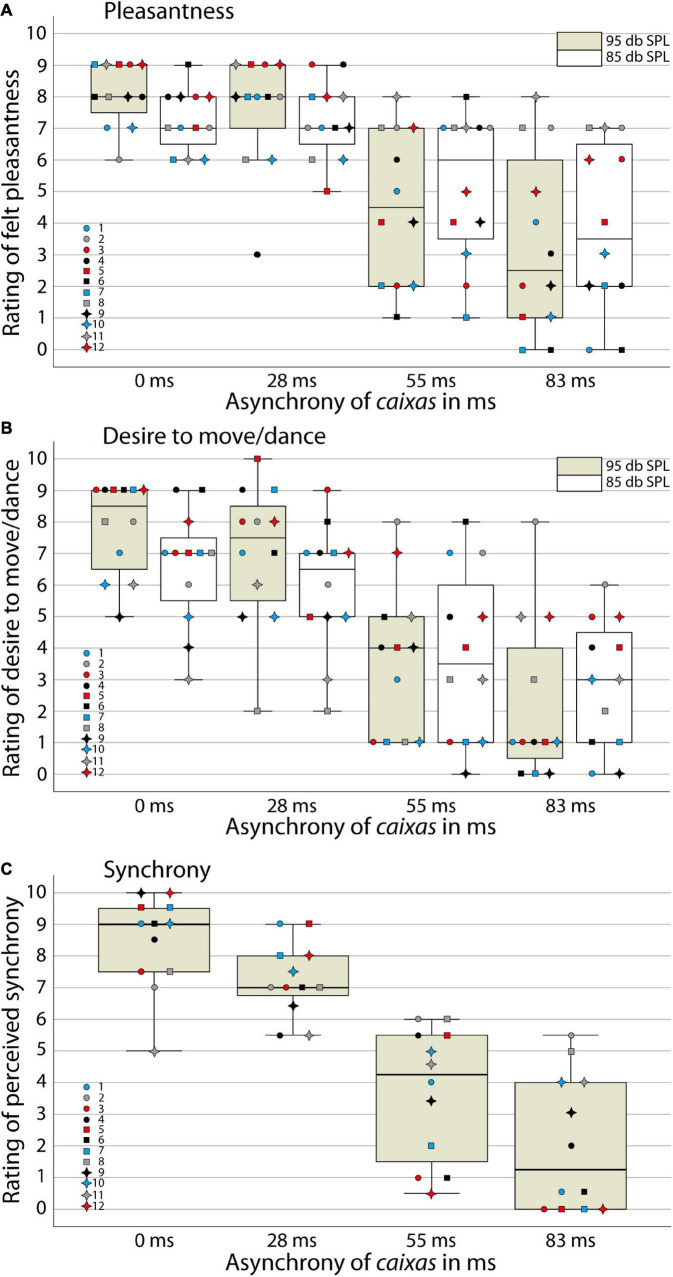
Ratings in the behavioral study. Boxplots with ratings of individual participants for **(A)** felt pleasantness, **(B)** the desire to move/dance and **(C)** perceived synchrony between instruments for samba percussion stimuli that varied in degree of asynchrony **(A–C)** and loudness **(A,B)**. The box indicates the interquartile distance with the first (lower end) and third (upper end) quartile. Whisker shows 1.5 times of the interquartile distance for indicating extreme values. Line indicates the median. Colored bar: very loud stimuli presented at 95 dB SPL, white bar: loud stimuli presented at 85 dB SPL. Colored data point symbols represent single participants.

We analyzed participants’ responses of the first perception task on their experienced pleasantness and their desire to move/dance with the samba percussion stimuli (Figures 2A,B) using a 2 (task: pleasantness/desire to move rating) × 2 (loudness: very loud/loud stimuli) × 4 (synchrony between instrumental parts: 0, 28, 55, or 83 ms delay of snare drums) ANOVA. We found a significant main effect of task [*F*_(1,11)_ = 4.9; *p* < 0.05, η^2^ = 0.31], indicating that participants generally felt more pleasantness with the stimuli than a desire to move/dance with the samba percussion sounds. A significant main effect of synchrony [*F*_(3,9)_ = 10.5; *p* < 0.01, η^2^ = 0.78] confirms our hypothesis that experienced pleasantness and the desire to move/dance with the samba percussion sounds decreased with increasing asynchrony. In order to explore the main effect of synchrony, the difference contrasts were examined. While the difference between 0 and 28 ms stimuli was not statistically significant [*F*_(1,11)_ = 2.2; *p* = 0.17, η^2^ = 0.17], the two other contrasts for 28 ms vs. 55 ms [*F*_(1,11)_ = 20.7; *p* < 0.01, η^2^ = 0.65] and 55 vs. 83 ms [*F*_(1,11)_ = 25.9; *p* < 0.001, η^2^ = 0.70] revealed significant differences. In addition, there was a significant interaction of loudness × synchrony [*F*_(3,9)_ = 4.1; *p* < 0.05, η^2^ = 0.58]. Inspection of descriptive statistics suggests that more synchronous stimuli (0 and 28 ms) evoked more pleasantness and a greater desire to move/dance when they were very loud compared to loud versions. When the stimuli contained larger asynchronies between instruments (55 and 83 ms), the lower loudness level tended to be rated as evoking more pleasantness and a greater desire to move/dance. To shed light on this interaction contrast, we merged data from the two tasks and calculated *t*-tests comparing very loud and loud versions of the stimuli for the different levels of synchrony. These analyses indicate that the very loud synchronous percussion sounds induced greater pleasantness and desire to move/dance than the loud versions [*t*_(11)_ = 3.4, *p* < 0.01, Bonferroni corrected for multiple testing, i.e., at the *p* < 0.0125 criterion], while there were no significant differences between very loud and loud versions of the asynchronous stimuli with delays between instrumental parts [28 ms *t*_(11)_ = 1.6, *p* = 0.15; 55 ms *t*_(11)_ < 1; 83 ms *t*_(11)_ = –1.1, *p* = 0.30]. There was neither a main effect of loudness [*F*_(1,11)_ < 1, η^2^ = 0.05] nor other significant interactions [task × loudness, *F*_(1,11)_ = 2.6; *p* = 0.14, η^2^ = 0.19; task × synchrony, *F*_(3,9)_ < 1, η^2^ = 0.17; task × loudness × synchrony, *F*_(3,9)_ < 1, η^2^ = 0.11].

For the second perception task, where participants evaluated synchrony between instruments explicitly (using only very loud stimuli; Figure 2C), an ANOVA analyzing the levels of synchrony confirmed a significant main effect [*F*_(3,9)_ = 13.4, *p* < 0.001, η^2^ = 0.82; with significant differences between all levels of synchrony, *p*s < 0.01, Bonferroni corrected] consistent with sensitivity to the asynchrony manipulation. Stimuli with more asynchrony between instrumental parts were evaluated with lower ratings of synchrony, i.e., perceived as played out of time.

#### Participants’ Rhythmic and Perceptual Abilities

In order to explore interindividual differences, two tests on measures of participants’ rhythmic and auditory perceptual abilities were analyzed. Performance on the Musical Ear Test (MET) ranged from 61.5 to 88.5% accuracy rate (mean ± SD: 79.6 ± 7.8%), i.e., well above chance (50%). Perceptual thresholds for perceiving two sounds as simultaneous had a range from 2.7 to 21.8 ms (mean ± SD: 8.0 ± 5.7 ms), i.e., below the levels of asynchrony in our manipulated stimuli.

We assumed that participants with better rhythmic and perceptual abilities would be better at detecting asynchronies between instrumental parts, as manipulated in our stimuli, and that this sensitivity should be reflected in participants’ ratings. Therefore, we calculated sensitivity indices by computing correlations between the (objective) values (0, 28, 55, 83) used to delay the snare drums in our stimuli and participants’ (subjective) ratings (ranging from 0 to 10) of (A) felt pleasantness and (B) desire to move/dance and (C) the direct evaluation of synchrony. Individual participants’ sensitivity index coefficients varied between *r* = 0.23 to –0.96 (mean ± SD: *r* = –0.62 ± 0.39) for the pleasantness ratings (A), and *r* = 0.06 to –0.92 (mean ± SD: *r* = –0.70 ± 0.31) for the ratings on the desire to move/dance (B), and *r* = –0.30 to –0.95 (mean ± SD: *r* = –0.83 ± 0.18) for the direct rating on synchrony (C). More negative values indicate greater sensitivity to the synchrony manipulation (i.e., stimuli with higher asynchrony between instrumental parts were given lower subjective ratings). The three sensitivity indices were in turn correlated with: (1) performance on the test on rhythmic abilities (MET range 61.5–88.5% correct answers); and (2) perceptual thresholds for perceiving two sounds as simultaneous (range 2.7–21.8 ms). Rhythmic abilities (performance in MET) were found to be related to the sensitivity indices based on pleasantness ratings (*r* = –0.57, *p* < 0.05) and ratings of the desire to move/dance (*r* = –0.55, *p* < 0.05). Specifically, participants who achieved better performance in the rhythmic abilities test rated more synchronous stimuli as being more pleasurable and expressed a stronger desire to move/dance. No such correlation was observed for the sensitivity index based on direct ratings of synchrony (C) and there were likewise no significant correlations between perceptual thresholds for perceiving sounds as being simultaneous and any of the three ratings-based sensitivity indices.

#### Questionnaire Responses

Participants indicated that they normally feel emotions such as joyful activation (rating mean ± SD: 4.5 ± 0.5 on a 5-point scale) and power (4.2 ± 1.3; see Supplementary Figure 1A for all ratings) when listening to samba percussion in daily life. Furthermore, participants reported usually having physiological sensations such as of goose bumps (3.8 ± 1.0) or a racing heart (3.1 ± 1.1) when listening to samba percussion (see Supplementary Figure 1B for all ratings).

### Functional Magnetic Resonance Imaging Study

#### Behavioral Measures

**Behavioral responses during functional magnetic resonance imaging scanning**: Confirming our objective categorization of stimuli, participants’ ratings on how pleasant was listening to the music excepts obtained during fMRI scanning revealed that listening to synchronous stimuli, i.e., when instruments were aligned “in time”, was most pleasant (mean ± SD: 8.9 ± 0.7). It was still pleasant to listening the asynchronous (“out of time”) stimuli with a 28 ms delay of the snare drums (8.4 ± 0.9). However, ratings of pleasantness dropped considerably for the stimuli that delayed the snare drums by 55 ms (4.5 ± 1.7) or 83 ms (2.7 ± 1.7). An ANOVA analyzing the levels of synchrony confirmed a significant main effect [*F*_(3,18)_ = 74, *p* = 0.001, η^2^ = 0.93]. Differences between all synchrony conditions were significant (*p*s < 0.001 for comparisons between conditions, Bonferroni corrected).

**Ability/sensitivity to detect asynchronies between instrumental parts**: In a behavioral test after fMRI scanning, participants were required to evaluate the synchrony between instrumental parts in the samba percussion stimuli on a scale ranging from 1 = “not at all synchronously” to 10 = “perfectly synchronized”. More synchronous stimuli were evaluated as being better synchronized (mean ± SD 0 ms: 9.1 ± 0.8; 28 ms: 8.2 ± 1.1) than stimuli that had larger timing shifts (55 ms: 3.6 ± 1.5; 83 ms: 2.3 ± 1.1). An ANOVA analyzing the judgments for different levels of synchrony confirmed a significant main effect [*F*_(3,18)_ = 134, *p* < 0.001, η^2^ = 0.96]. The differences between all levels of synchrony were significant (*p*s < 0.001 for comparisons between conditions, Bonferroni corrected).

In order to assess participants’ abilities to perceive different degrees of synchrony in the samba percussion stimuli, we calculated an index of the sensitivity to timing shifts, as in the behavioral study, by correlating the (objective) asynchrony values (0, 28, 55, 83 ms) with the participants’ (subjective) ratings. More negative values reflect higher concordance between perception of synchrony and objective synchrony, with *r* = –1.0 indicating a perfect match. Correlation coefficients ranged from *r* = –0.93, indicating high sensitivity to different levels of synchrony, to *r* = –0.55, indicating moderate sensitivity to asynchronies (mean ± SD: *r* = –0.84 ± 0.10).

**Questionnaires**: Responses in the questionnaire about participants’ general emotional experience (GEMS-9, Zentner et al., 2008) when listening to samba percussion in daily life indicate that participants normally feel emotions such as joyful activation (rating mean ± SD: 4.4 ± 0.7 on a 5-point scale) and power (4.0 ± 1.0) as well as wonder (4.0 ± 1.1; see Supplementary Figure 2A for all ratings). Furthermore, participants reported usually having physiological sensations such as goose bumps (3.8 ± 1.1) and a racing heart (3.5 ± 1.3) when listening to samba percussion (see Supplementary Figure 2B for all ratings).

With regard to identifying “deep listeners”, 14 participants reported that their emotional responses when listening to the samba percussion in daily life are stronger (*n* = 9) or much stronger (*n* = 5) than the corresponding emotional responses of most people they know, and seven participants indicated that their emotional responses are weaker than (*n* = 1) or equal to (*n* = 6) others’ emotional responses. Evaluations of participants classified as “deep listeners” or “non-deep listeners” on emotional and physiological responses when listening to samba percussion (and several other ratings) can be found in Supplementary Material (2.3 Description of deep listeners and non-deep listeners by their self-reported evaluations, Supplementary Figure 3). Participants who considered themselves to have stronger emotional responses when listening to samba percussion (“deep listeners”) reported feeling more joyful activation, wonder, transcendence and nostalgia as well as more physiological sensation of a “racing heart” (cf. Supplementary Figure 3) compared to participants (*n* = 7) who indicated that their emotional responses are weaker than or equal to others’ emotional responses (non-deep listeners).

#### Functional Magnetic Resonance Imaging Data

**FIGURE 3:**
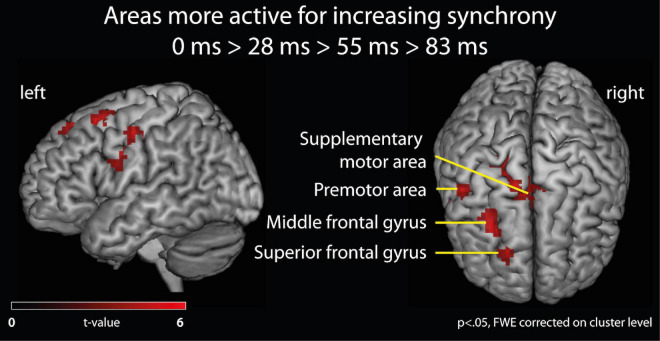
fMRI analysis of brain activation. Brain areas active for listening to samba percussion stimuli with varying degrees of synchrony between instruments. Contrast shows increasing brain activation with increasing synchrony between instruments in the ensemble at *p* < 0.05, FWE corrected at cluster level.

**Effects of varying degrees of synchrony in samba percussion**: In order to identify which brain areas were more active for synchronous samba percussion, we calculated a contrast testing for brain responses showing increased activation when listening to stimuli with increasing synchrony between instruments (i.e., the contrast for 0 > 28 > 55 > 83 ms). Thus, significant activations in this analysis reflect stronger brain activity for stimuli that were more “in time” (i.e., with lower asynchrony between instruments). This analysis revealed stronger hemodynamic responses in the supplementary motor area (SMA proper) that were more pronounced in the left hemisphere, but also extended into the right hemisphere, the left middle frontal gyrus partly extending into the superior frontal gyrus (cluster covering Brodmann area, BA, 6 and 8), and the left premotor areas (BA6) extending into the primary motor (BA4) and somatosensory area (BA3). These areas showed significant results when correcting for multiple comparisons (*p* < 0.05, family-wise error, FWE, corrected on a cluster level, Table 1 and Figure 3).

**TABLE 1:** Brain areas that show stronger activation for increasing synchrony (contrast: 0 > 28 > 55 > 83 ms).

Anatomical region	Hemisphere	MNI coordinates			Cluster size	Z-score
		*x*	*y*	*z*		
***p* ≤ 0.05, Family Wise Error (FWE) corrected on the cluster level**						
SMA	L	−12	−16	68	*381*	*4.50*
MFG (BA6/8)	L	−30	17	56	*159*	*4.12*
Superior frontal gyrus		−18	35	53		
Premotor area	L	−51	−10	47	*84*	*3.93*
IFG (pars opercularis)		−60	5	23		
**Exploratory analysis at *p* < 0.005, uncorrected, cluster size ≥ 10 voxels**						
***Frontal areas***						
Premotor area	R	27	−25	68	69	3.80
Premotor area (dorsal)	R	48	−16	56	39	3.59
Premotor area (dorsal)	R	33	−19	56	11	3.25
Premotor area	R	54	−7	41	12	2.82
Rolandic operculum	L	−48	−1	8	17	3.18
IFG pars triangularis	L	−36	35	14	10	3.82
Middle frontal gyrus	R	30	23	50	18	3.25
***Parietal areas***						
Somatosensory area (BA3/SII)	R	57	−4	17	46	3.89
Inferior parietal lobe	L	−42	−67	38	16	3.23
***Temporal areas***						
Hippocampus	R	18	−10	−25	23	3.71
Hippocampus	L	−18	−13	−19	56	3.44
Amygdala		−21	2	−16		2.87
Middle temporal gyrus	L	−39	5	−28	11	3.56
Middle temporal gyrus	L	−63	−10	−22	14	3.05
Middle temporal gyrus	L	−48	−61	−4	12	2.83
Fusiform gyrus	L	−45	−55	−16	27	3.27
***Occipital areas***						
Middle occipital gyrus	L	−30	−70	38	28	3.57
***Medial areas***						
MCC	L	−3	−31	38	15	3.62
MCC	R	18	−19	44	16	2.92
***Cerebellum***						
Cerebellum Lobule VII (Hem.)	R	45	−70	−40	28	3.78
***Other areas***						
Putamen/pallidum	R	27	2	1	17	3.67
Putamen	R	27	−10	8	11	3.27
Cluster reaching into pallidum and putamen	L	−18	2	−7	12	3.07
Thalamus	L	−24	−19	14	16	2.95

*The values shown are Montreal Neurological Institute (MNI) coordinates (x, y, z) for significant activation maxima of clusters in the random effects analyses with a minimum cluster size of 10 voxels. IFG, inferior frontal gyrus; SII, secondary somatosensory area; Hem., Hemisphere; BA, Brodmann area; R, right; L, left; SMA, supplementary motor area; MFG, Middle frontal gyrus; MCC, middle cingulate cortex.*

In order to further explore the fMRI data, we report brain areas showing effects for the same contrast at an uncorrected significance level (*p* < 0.005, minimum cluster size of 10 voxels). The following activation clusters were found (see Table 1): right premotor area extending into the primary motor (BA4) and somatosensory area (BA3, BA1); right somatosensory area (BA3) extending into secondary somatosensory area (SII) and primary motor cortex; left rolandic operculum; right inferior frontal gyrus (pars triangularis); right middle frontal gyrus; left inferior parietal lobe (BA39); a cluster in the right cerebellum (lobule VIIa crus I hemisphere); bilateral putamen and hippocampal region (subicular complex, entorhinal cortex, cornu ammonis) extending into the amygdala; bilateral middle cingulate cortex; left middle occipital gyrus; left middle temporal gyrus and fusiform gyrus; left thalamus. Because the subgenual cingulate cortex is a small structure and a region of interest for further analysis, it can be mentioned that there is a small cluster (6 voxels, *x* = –3, *y* = 17, *z* = –10, *Z* = 2.93) at this exploratory significance level of *p* < 0.005.

In order to explore brain responses in the nucleus accumbens (as key structure of the reward network), we performed a region of interest analysis using an anatomical mask (Pauli et al., 2018) and found no significant results between experimental conditions (see Supplementary Figure 4).

The reverse contrast exploring brain areas that show stronger activity for increasing asynchrony in samba percussion stimuli revealed no significant brain activations in motor-related areas when correcting for multiple comparisons. The brain areas revealed in an exploratory analysis at a lower, uncorrected significance level are listed in Supplementary Table 2.

#### Combined Behavioral and Functional Magnetic Resonance Imaging Results: Exploratory Analyses on Interindividual Differences

**FIGURE 4:**
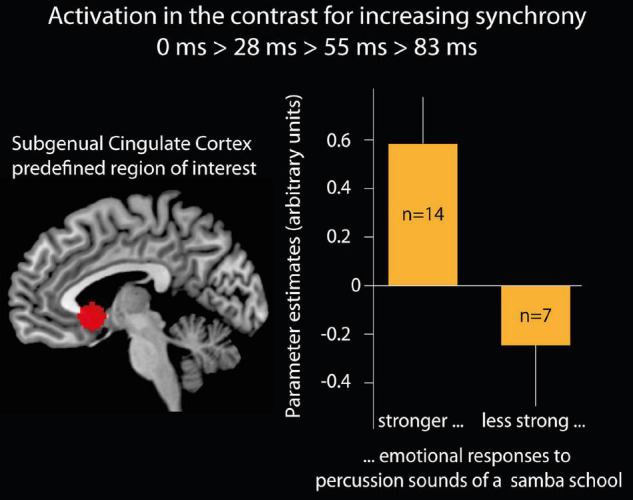
Brain activation of the subgenual cingulate cortex. Left side: region of interest (ROI) of the subgenual cingulate cortex defined as a 10 mm sphere at a predefined coordinate (Moll et al., 2006; *x* = –2, *y* = 15, *z* = –5). Right side: bar graphs show the mean and standard error of the mean for the parameter estimates (arbitrary units) in the subgenual cingulate cortex ROI for the contrast of increasing synchrony between instruments. The bars present 14 participants who believed that their emotional responses when listening to samba percussion in daily life are stronger than the emotional responses of most of the people they know and seven participants who indicated that their emotional responses are less than or equal to most of the people they know.

**Relations with emotional responses to samba percussion in general**: Parameters for brain activation from the contrast 0 > 28 > 55 > 83 ms were extracted for the subgenual cingulate cortex (region of interest defined as a 10 mm sphere at a predefined coordinate, Moll et al., 2006: *x* = –2, *y* = 15, *z* = –5) and compared between groups of participants who were classified as deep listeners versus non-deep listeners. Participants who reported stronger emotional responses when usually listening to samba percussion (deep listeners) showed stronger brain activation (Parameter estimates, PE, mean = 0.58, SD 0.71, s.e.m. 0.19) than participants with weaker emotional responses [non-deep listeners: PE = –0.26, SD 0.67, s.e.m. 0.25; *t*(19) = 2.6, *p* < 0.05, two-tailed, see Figure 4]. In order to test whether this effect was specifically related to experiencing pleasantness, we analyzed the brain response in the reward area of the nucleus accumbens using an anatomical mask (Pauli et al., 2018) and found no differences (“deep listener” PE = 0.41, SD 0.65, s.e.m. 0.17, “non-deep listener” PE = 0.20, SD 0.80, s.e.m. 0.33; *t* < 1).

**Sensitivity to differences in degree of synchrony**: For this analysis, we used the behavioral index of sensitivity to timing shifts (i.e., the correlation between the objective values and subjective ratings for perceived synchrony acquired after fMRI scanning) in a parametric analysis of the fMRI data. Specifically, we tested whether fMRI effects for the contrast 0 > 28 > 55 > 83 ms, which showed increasing brain activation for increasing synchrony, is related to the individual differences in sensitivity to asynchronies. No significant results were found when correcting for multiple comparisons (*p* < 0.05, FWE, corrected on a cluster level). However, given that the sample size of 21 participants is rather small and homogenous in terms of musical experience (and our sample did not include non-musicians or participants with very poor rhythm perception abilities), we conducted an exploratory analysis of individual differences with uncorrected significance levels (*p* < 0.005, minimum cluster size of 10 voxels). This analysis revealed relations between a better ability to detect asynchronies in the behavioral test and hemodynamic responses for the contrast of increasing synchrony in instrumental parts (0 > 28 > 55 > 83 ms) in the left cerebellum and the temporal lobe/amygdala (see Supplementary Table 3).

## Discussion

In the current research, we investigated the effect of varying degrees of (a)synchrony between multiple instrumental parts in samba percussion music on listeners’ groove-related subjective experiences and associated brain responses. First, experiences of pleasantness and the desire to move/dance were assessed for samba percussion stimuli with varying degrees of asynchrony presented at loud or very loud sound intensity levels in a behavioral experiment. Second, in an fMRI study, neural processing was examined in amateur musicians as they listened to the same stimuli with varying levels of synchrony (at only one loudness level) and rated how pleasant was listening to the excerpt. In both studies, interindividual differences related to auditory perceptual abilities and emotional responsiveness to samba percussion were examined in an exploratory manner. Listeners’ subjective ratings indicated that increasing synchrony between instrumental parts was associated with greater experienced pleasantness in both studies, and with the increasing desire to move/dance in the behavioral study. Moreover, the fMRI results indicated that listening to samba percussion with increasing synchrony was accompanied by stronger brain activity in the supplementary motor area bilaterally, the left middle frontal gyrus, and the left premotor area. Furthermore, participants who reported having stronger emotional responses when listening to samba percussion in daily life displayed stronger brain activations in the subgenual cingulate cortex. Additionally, in the behavioral study, we found that participants with better rhythmic discrimination abilities were more sensitive to the synchrony manipulation in the stimuli. Below we discuss additional interindividual differences in neural processing related to perceptual sensitivity (observed at an uncorrected significance level) in the expectation that they will be useful for generating hypothesis in future studies.

### Behavioral Effects of Varying Synchrony in Samba Percussion and Sound Intensity

We show in both the behavioral study and the behavioral measures of the fMRI experiment that individuals who are familiar with the rhythm of samba percussion music were sensitive to our objective manipulation of synchrony between instrumental parts, which was in a range (28–83 ms) that corresponds to asynchronies commonly observed in ensemble performance (Keller, 2014). The results of our behavioral study demonstrated that percussion stimuli with high synchrony between instrumental parts evoked more pleasantness and a greater desire to move/dance than stimuli with lower synchrony, suggesting a link with the concept of “feeling of groove” (cf. Witek et al., 2014; Matthews et al., 2019, 2020). Synchrony between instruments, or in other words “playing in time”, may thus contribute to the experience of “groove” in ensemble music, possibly by modulating beat salience (Keil, 1995; Hove et al., 2007; Madison et al., 2011).

The behavioral results on experienced pleasantness obtained with samba-experienced listeners in both of our studies are consistent with other studies showing effects of familiarity on groove ratings (Senn et al., 2018), as well as effects of asynchronies in percussion sounds, especially when the snare drums were shifted in time (Frühauf et al., 2013; Senn et al., 2017). Corroborating this, Frühauf et al. (2013) found that judgments of drum pattern quality (assessed by different criteria including ratings about liking and feeling animated to move for drum patterns played on a snare and a bass drum) were higher for synchronized (i.e., quantized) stimuli than for stimuli with increasing deviations in microtiming (15 and 25 ms shifts of either instrument from the beat). In the stimuli for the current studies, the snare drum was shifted in time relative to other instruments in a fixed manner, i.e., resulting in a constant degree of overall asynchrony throughout an excerpt, while leaving the musical structure intact. Such fixed shifts differ from variability in timing associated with increasing expressiveness, as such variability is applied locally, flexibly and not constantly in terms of magnitude (Clarke, 1989; Keil, 1995). In the present studies, expressive timing variations were inherent in the natural recordings of the professional musician who produced the stimuli (see section “Materials and Methods” and Supplementary Material). Our experimental manipulation of asynchronies with a fixed shift of one part led to a less pleasant experience, which is consistent with Senn et al. (2017), who compared fixed and flexible time shifts. Although constant shifts may seem unnatural or “mechanical”, in real life, the spatial distance between co-performers, including instrument groups in street parades during carnival, leads to sound transmission delays that challenge musical ensemble coordination by introducing time shifts between instruments (Bartlette et al., 2006).

In our behavioral study, we additionally investigated effects of sound intensity. Confirming our hypothesis, we found that very loud (95 dB SPL) samba percussion sound evoked more pleasantness and greater desire to move/dance than loud (85 dB SPL) sounds, but only when the sounds were well synchronized across parts. A possible explanation for these effects is that very loud percussion sounds elicit a more pronounced vestibular response, in addition to auditory responses, that enhance emotional processing (Todd et al., 2000; Todd and Lee, 2015; Rajagopalan et al., 2017). Consistent with this interpretation, Todd and Cody (2000) found that samples of loud techno music (above 90 dB SPL) elicited a greater vestibular responses associated with pleasurable self-motion. Also in alignment with our results, Hove et al. (2020) found that listeners gave music clips higher groove and enjoyment ratings when the clips were relatively loud (see also Hove et al., 2019 for effects of loudness) and when auditory presentation was accompanied by additional tactile stimulation. However, in contrast to these findings, Stupacher et al. (2016) reported that sound intensity had no effect on groove ratings. In the relevant study, participants listened to music excerpts with high, medium, and low groove, presented with high (0 dB), mid (–6 dB), and low (–12 dB) sound intensities, and were asked to rate on a 7-point scale “to what extent did you feel that the musical excerpt grooved” (Stupacher et al., 2016, study 2). Their results showed an effect of groove levels on ratings, but there was neither a main effect of sound intensity nor an interaction of sound intensity with different levels of groove on ratings by the participants. Their findings are consistent with Janata et al. (2012), who report that listeners did not agree with the statement “The groove depends on the overall loudness of the music”. In our studies, we had no direct rating for groove, but instead asked listeners about felt pleasantness (in both studies) and the desire to move/dance when listening to our music excerpts (in our behavioral study). Our results of the behavioral study therefore support the assumption that loudness alone does not increase pleasurable experience for percussion stimuli, but rather that such experiences also depend on musical features such as synchrony between instrumental parts, which might specifically influence beat salience (i.e., not just overall sound salience).

### Neural Correlates of Varying Degrees of Synchrony in Samba Percussion

Analyses of fMRI data focused on contrasting brain activations while listening to the varying degrees of asynchrony in the samba percussion stimuli. The results revealed that listening to stimuli with higher synchrony between instrumental parts was accompanied by stronger brain activation in motor-related areas including the supplementary motor area and premotor area, as well as the middle frontal gyrus (cluster covering areas of BA6 and 8), despite the fact that participants listened passively (without overt movement). This finding is broadly consistent with research (reviewed in the introduction) that points to the involvement of motor-related areas during perceptual processing (Hommel et al., 2001; Rizzolatti and Craighero, 2004; Schubotz, 2007) and more specifically in beat perception and rhythm processing (Grahn and Brett, 2007; Chen et al., 2008; Bengtsson et al., 2009; Grahn and Rowe, 2009, 2013; Todd and Lee, 2015; Matthews et al., 2020). Activity in these motor-related areas (especially the supplementary motor area, premotor cortex, basal ganglia, and cerebellum) is assumed to be functionally linked to internal representations of the beat that play a role in forming temporal predictions and musical expectations by recruiting prefrontal and parietal areas (Schubotz, 2007; Novembre and Keller, 2014; Vuust and Witek, 2014; Morillon and Baillet, 2017; Vuust et al., 2018; Koelsch et al., 2019). This view is consistent with the proposal that there is a dorsal stream of auditory processing that plays a role in sensorimotor coupling and prediction (Rauschecker, 2018). The dorsal stream comprises a route stemming from primary auditory areas over the inferior parietal lobe to premotor areas (BA 6 and 8), terminating in the inferior frontal gyrus (BA44). The present finding of stronger activity in the supplementary motor area, premotor area, and middle frontal gyrus (BA6/8), is in agreement with this proposed role of sensorimotor coupling in prediction (beat representation and forming rhythmic expectations). Stimuli where percussion instruments play “in time” presumably facilitate these prediction processes, as compared to stimuli where time shifts in instrument groups increase temporal complexity, due to greater clarity in signaling the underlying beat structure. This interpretation is consistent with evidence that pulse clarity is associated with strengthened audio-motor coupling network (Burunat et al., 2017). Our exploratory results (at an uncorrected significance level) revealed an extended network of additional motor areas including basal ganglia and the cerebellum, further corroborating the interpretation that audio-motor coupling was strengthened when listening to more synchronized stimuli.

In the domain of music perception generally, the coupling between auditory and motor areas is especially relevant to the extent that the strength of such coupling increases with musical expertise (Bangert et al., 2006; Lahav et al., 2007; Mutschler et al., 2007; Zatorre et al., 2007; Engel et al., 2012). Although we did not compare musicians and non-musicians, the fact that most of participants in our fMRI study had expertise in playing samba percussion pattern fits with previous findings that action expertise contributes to the level of processing in motor-related areas during perception (Bangert et al., 2006; Lahav et al., 2007; Zatorre et al., 2007). The strength of audio-motor coupling in the brain also has behavioral consequences through effects on movement execution. Janata et al. (2012) studied groove as a sensorimotor phenomenon, and found that high groove music yielded more accurate movement synchrony in a tapping task, as well as inducing a greater amount of spontaneous movement, such as foot tapping (see also Witek et al., 2014). Furthermore, Leow et al. (2014, 2021) highlighted implications of audio-motor coupling for gait rehabilitation in clinical settings by showing that high-groove music elicited longer and faster steps than low-groove music in healthy adults (see also Hove and Keller, 2015). Trost et al. (2017) discussed how rhythmic entrainment contributes to induce (positive) musical emotions by differentiating several levels of possible entrainment mechanisms (on neural, perceptual, autonomic physiological, motor, social, and subjective levels). Stronger audio-motor coupling while listening to samba percussion with high synchronization between instruments might trigger entrainment on several levels that in turn contribute to the desire to move and evoke pleasantness, with these multilevel processes collectively leading to the experience of “feeling the groove”. The present results thus add to knowledge on the brain bases of music processing by demonstrating that synchronization between instruments might influence feeling the groove, most likely by modulating beat salience, predictive processing, and multilevel entrainment.

Experiencing pleasurable music activates brain areas implicated more broadly in reward processing (Blood and Zatorre, 2001; Koelsch et al., 2006; Koelsch, 2010; Trost et al., 2012). These areas include the striatal dopaminergic system (Salimpoor et al., 2011, 2013; Zatorre and Salimpoor, 2013), which is involved in peak emotional responses marked by “chills” (Panksepp, 1995; Grewe et al., 2007, 2009; Salimpoor et al., 2009; see Witek, 2009 for peak emotional responses/physiological arousal when experiencing groove). Also, Matthews et al. (2020) report activation of reward areas for music excerpts that are perceived to have higher groove. However, we failed to find reward-related brain activity when listening to samba percussion stimuli with greater synchrony between instruments. Specifically, we found no evidence for significantly stronger activation in the core structure of the reward network, i.e., the ventral striatum, although some of the brain areas at lower levels of significance might be considered as being part of a more extended reward network (e.g., putamen, amygdala, hippocampus, medial areas). In an additional region of interest analysis (Supplementary Figure 4), we found a trend for higher activity in the nucleus accumbens for samba percussion with higher synchrony between instruments, together with high variability between participants (which presumably contributed to non-significant results). Several explanations may account for this outcome.

First, it is unlikely that listening to the synchronous samba percussion in our study reliably evoked an emotional peak response (“chill”), which could, under other circumstances, lead to an activation in the nucleus accumbens (Salimpoor et al., 2011). We used a block design with repetitions and our stimuli did not contain much rhythmic variation within the 20 s excerpt. More extensive musical context and greater variation of musical (rhythmical) parameters might be necessary to reliably evoke peak emotional responses like “chills”. Such peak responses, together with activity in the nucleus accumbens, might be better captured with event-related designs where activity is locked to changes in stimuli (e.g., events that evoke surprise due to violated expectations) and which also capture phasic responses. Our stimuli were not constructed with the aim of violating expectations or evoking peak emotional responses. In the context of groove, a so-called “groove listening state” rather than peak-based emotional response has been postulated, consistent with subjective descriptions by listeners (Witek, 2009).

A second factor that could have contributed to the lack of direct evidence for reward processing relates movement constraints. Listening to high groove music elicits the desire to move/dance along—as our stimuli did (cf., the behavioral study). However, any movement, even finger tapping, was restricted during the fMRI experiment. Indeed, as debriefing of participants confirmed, not moving was difficult and frustrating for some of the participants. Thus, a main aspect of feeling the groove, namely moving with the beats, was eliminated, which might have reduced the pleasurable experience. Third, over the course of the experiment, participants listened to similar stimuli repeatedly, which might have caused habituation of brain responses, thus diminishing pleasurable experiences and related brain responses.

### Exploratory Results on Interindividual Differences in Neural Processing

Previous studies have shown that being in synchrony with others while performing collective musical actions (e.g., singing, finger tapping or drumming together) increases trust, affiliation, and prosocial behavior (Anshel and Kipper, 1988; Hove and Risen, 2009; Kirschner and Tomasello, 2009; Wiltermuth and Heath, 2009; Kokal et al., 2011). The present research addressed *the perception* of synchrony in such musical actions, under the assumption that listening to samba percussion, which requires high interpersonal synchronization (i.e., high synchrony among instrumental parts or playing “in sync”), recruits similar brain areas as executing such synchronous actions (Hommel et al., 2001; Rizzolatti and Craighero, 2004; Schubotz, 2007). These include motor areas, reward areas, and also brain areas related to prosocial behavior and further emotional processing linked to affiliation and feeling connected with others. Individual differences become relevant to the extent that the level of activation in these brain areas might be related to the concept of “deep listeners” (Becker, 2004), defined here as individuals who have pronounced emotional experiences and physiological sensations when listening to music or percussion in general (see also Chapin et al., 2010). Such emotional responses to music on a trait level might also contribute to feeling connected with others easily, social bonding and prosocial behavior when experiencing synchrony in musical ensembles. In the sample in our fMRI study, most participants were classified as “deep listeners”, as they described themselves as having stronger emotional responses when listening to samba percussion relative to people they know. Descriptive data included in Supplementary Figure 3 confirm differences in emotional and physiological experiences while listening to samba percussion, according to self-reported ratings. Although we have unequal groups (14 deep listeners and 7 non-deep listeners) in our fMRI study and low power in terms of sample size, we nonetheless analyzed brain responses in the subgenual cingulate cortex as a region of interest as intersection for emotional experience and social bonding in order to explore inter-individual differences. The subgenual cingulate cortex was found to respond more strongly in “deep listeners”. Thus, merely perceiving synchrony in musical actions leads to differential activation of a brain area that has been implicated in social processing related to attachment, affiliation, prosocial behavior, altruistic decisions, ingroup related effort, and belongingness to a group (Bartels and Zeki, 2004; Aron et al., 2005; Moll et al., 2006, 2012; Krueger et al., 2007; Zahn et al., 2009; Rusch et al., 2014; Bortolini et al., 2017). Furthermore, the subgenual cingulate cortex is a key hub in emotional processing, and a target for deep brain stimulation in treatment resistant major depression disorder (Kibleur et al., 2017). Although our findings on this issue are preliminary, they invite further investigation of the effects of emotional processing and entrainment during both active and passive participation in joint music-making on prosocial behavior toward co-participants (see also Kokal et al., 2011; Trost et al., 2017). Our results likewise encourage the future exploration of therapeutic uses of music with groove-inducing properties in behavioral and neuropsychiatric disorders characterized by the impaired functioning of neurophysiological mechanisms related to entrainment and social and emotional processing (Hove and Keller, 2015; Koelsch, 2015; Aalbers et al., 2017; Braun Janzen et al., 2019).

In such future work, the concept of “deep listeners” deserves greater scrutiny. To classify participants as “deep listeners” in the current work, we used participants’ responses to a question probing whether their emotional responses, when listening to samba percussion in general, are stronger than in most other people they know (cf. Becker, 2004; Chapin et al., 2010). Future studies that focus on interindividual differences in emotional experience with music might benefit from using objective measures to classify participants. For example, physiological responses to stimuli could be recorded and used instead of self-evaluations of emotionality (possibly in more naturalistic or simulated virtual environments to capture social aspects). Alternatively, composite scores based on several different emotions felt in response to music could be used.

In addition to interindividual differences related to social and emotional processing, our results yielded evidence for individual variation related to more basic perceptual-motor processing capacities. Specifically, in our behavioral study, we found some indication that participants who had better rhythm perception abilities (measured by the MET, Wallentin et al., 2010) were more sensitive to asynchronies in the samba percussion stimuli, as reflected in ratings about felt pleasantness and the desire to move/dance. This finding motivated us to explore interindividual differences in brain responses in the fMRI experiment. Although our participants were musically trained, they varied in their ability to detect asynchronies in the samba percussion stimuli (reflected by the correlation of the objective asynchrony values in the stimuli and subjective ratings of synchrony obtained in the behavioral test after fMRI scanning). A parametric analysis that included this index of sensitivity revealed (albeit at a weak statistical level) that increasing synchrony was associated with stronger brain activity in motor related areas including the cerebellum, as well as in the temporal lobe/amygdala, to the greatest degree for participants who were better at detecting asynchronies. The cerebellum is involved in temporal processing of events not only in sensorimotor control, but also in perception, with a specific role in generating internal representations of temporal relations in the sub-second range (Spencer and Ivry, 2013; Sokolov et al., 2017). For example, patients with cerebellar lesions display decreased perceptual sensitivity to rhythmic perturbations in auditory sequences at beat rates relevant to musical tempo (Schwartze et al., 2016). Furthermore, in our fMRI study, we observed stronger responses in the amygdala for participants who were sensitive to different levels of synchrony. The amygdala has been found to be involved in a variety of auditory and musical tasks, e.g., during the perception of emotionally neutral stimuli that are ambiguous or difficult to predict in terms of their timing (Hsu et al., 2005; Herry et al., 2007). Amygdala activation during music listening has been was observed in response to the violation of listeners’ (harmonic) expectancies (Koelsch et al., 2008), dissonance (Trost et al., 2015), or when experts were listening to improvised melodies that contained relatively pronounced cues to behavioral uncertainty in the musical performance (Engel and Keller, 2011). Our findings thus indicate that participants who were better able to detect asynchronies in the samba percussion stimuli had stronger brain activation in brain areas involved in processing timing cues. These results are in line with previous studies investigating interindividual differences in beat perception and related brain activity, e.g., strong and weak beat perceivers (Grahn and Brett, 2007) and differences in musicians vs. non-musicians when listening to groove-based music (Matthews et al., 2020). However, our exploratory findings on this issue should be interpreted with caution, and need to be confirmed in future studies with larger sample sizes and greater variation in rhythmic abilities (e.g., by including non-musicians or individuals with weak beat perception capacities).

## Conclusion

The results of the present behavioral and fMRI studies indicate that listening to samba percussion music in which instruments are more “in time” leads to a greater desire to dance/move (in the behavioral study) and greater experienced pleasantness (in both studies), and that these experiences are accompanied by an activation of motor-related brain areas involved in rhythm processing and beat perception (Grahn and Brett, 2007; Chen et al., 2008; Bengtsson et al., 2009; Grahn and Rowe, 2009, 2013). Such motor-related brain activity for better synchronized stimuli might reflect a strengthening of audio-motor coupling that: (a) could be a basis for facilitating the desire to move/dance with the percussion sounds that have a predictable, strong beat as confirmed by the ratings of the behavioral study (cf., Janata et al., 2012; Stupacher et al., 2013; Matthews et al., 2020); and (b) leads to a pleasurable experience via entrainment operating at multiple levels (cf., Trost et al., 2017). With regard to individual differences, exploratory analyses suggested that listeners who reported stronger emotional responses to samba percussion in general showed higher brain activation in the subgenual cingulate cortex, an area that has been implicated in prosocial behavior, emotional processing, and attachment (Moll et al., 2006; Zahn et al., 2009). The role of this brain region, together with the more extended audio-motor network, in the prosocial effects of entrainment during joint music-making deserves further investigation (Kirschner and Tomasello, 2009; Kokal et al., 2011), especially insofar as it has implications for clinical uses of music in behavioral and neuropsychiatric therapy.

## Data Availability Statement

The raw data supporting the conclusions of this article will be made available by the authors on request, without undue reservation to any qualified researcher.

## Ethics Statement

The studies involving human participants were reviewed and approved by the Scientific Ethics Committee of Copa D’Or Hospital, Rio de Janeiro, Brazil. The participants provided their written informed consent to participate in this study.

## Author Contributions

AE, JM, PK, and SH designed the behavioral and fMRI study. AE, SH, and MM acquired the data of the behavioral and fMRI study. AE and SH analyzed the data with advice from and consultations with JM and PK. AE wrote the manuscript with contributions from all authors.

## Conflict of Interest

The authors declare that the research was conducted in the absence of any commercial or financial relationships that could be construed as a potential conflict of interest.

## Publisher’s Note

All claims expressed in this article are solely those of the authors and do not necessarily represent those of their affiliated organizations, or those of the publisher, the editors and the reviewers. Any product that may be evaluated in this article, or claim that may be made by its manufacturer, is not guaranteed or endorsed by the publisher.

## Footnotes

1 ***Background to Brazilian samba schools:*** A Brazilian samba school is an association that typically comprises hundreds of people involved in dancing, drumming (as well as playing percussion and other instruments), and singing during well-rehearsed carnival parades featuring original costumes and vehicles, following a specific theme and rules governing competition with other schools. The percussion section (*“Bateria de escola de samba”*) comprises about 250–300 percussionists in top samba schools in Rio de Janeiro. See section “Materials and Methods”, Figure 1 and Supplementary Material for a description of instruments and the typical rhythm. See here for a live recording: https://www.youtube.com/watch?v=QgXsUZHaYc4 and https://www.youtube.com/watch?v=Adw8u6gL2HE for a demonstration of single instruments in a Brazilian samba school percussion section. Being a member or fan of a particular samba school in Brazil contributes to social identity.

2
http://www.sph.sc.edu/comd/rorden/mricron/

3
http://www.fz-juelich.de/inm/inm-1/spm_anatomy_toolbox/

4
http://www.bobspunt.com/bspmview/

5
http://marsbar.sourceforge.net/

## Supplementary Material

Supplementary Audio 1 Example of a 20-s excerpt of samba percussion with 0 ms time shift of snare drums.

Supplementary Audio 2 Example of a 20-s excerpt of samba percussion with 28 ms time shift of snare drums.

Supplementary Audio 3 Example of a 20-s excerpt of samba percussion with 55 ms time shift of snare drums.

Supplementary Audio 4 Example of a 20-s excerpt of samba percussion with 83 ms time shift of snare drums.
