# Supplementary material for: Neural Correlates of Listening to Varying Synchrony Between Beats in Samba Percussion and Relations to Feeling the Groove
Source: Front Neurosci. 2022 Feb 25;16:779964. doi: 10.3389/fnins.2022.779964 (PMC8915847; doi:10.3389/fnins.2022.779964)
Supplement: Supplementary file 1 [file Data_Sheet_1.pdf]

Supplementary Material

1 Supplementary Methods

1.1 Participants

Behavioral experiment: 12 volunteers (mean age ± standard deviation, SD: 30.6±6.9 years, range: 20-40 years, 8 female) participated in the study. 8 of the 12 participants played one or more instrument(s). Participants musical experience was assessed by self-report of practice hours alone on their instruments (summed over instruments: years x hours per week x 52): 1700±2514 (range: 78-7800) hours alone on the instrument(s) and hours spent playing in musical ensembles: 1755±2010 (range: 234-5980). All participants liked (4.1±.8) listening to the percussion ensemble of a samba school and were familiar with its sound (3.8±.8) and the rhythm (3.8±1.0; values indicate mean and SD for ratings on scales ranging from 1='not at all' to 5='very much').

fMRI experiment: 24 volunteers participated in this fMRI study. 3 data sets could not be included in the analyses due to insufficient data quality (e.g., extensive head movements, frontal signal dropout due to some dental material). The final sample comprised 21 volunteers (mean age ± SD: 34.4±5.5 years; range 26 – 42 years, five female). All of them had musical experience, 5 of the 21 volunteers were professional and 16 amateur musicians. All participants played an instrument (e.g., 20 out of 21 participants played a percussion instrument or drums; 15 out of 21 participants played acoustic guitar, 6 out of 21 participants played piano). Participants musical experience was assessed by self-report of practice hours alone on their instruments (summed over instruments: years x hours per week x 52): 4822±6203 (range: 0-22750) and hours spent playing in musical ensembles: 4639±9867 (range: 5-43680) regardless style or genre. All participants liked (4.4±0.7) listening to samba percussion and were familiar with its sound (4.4±0.7) and rhythm (4.5±0.7; values indicate mean and SD for ratings on scales ranging from 1='not at all' to 5='very much'). 19 out of 21 participants reported that they could play the typical rhythm of samba percussion and 12 of them already participated in a percussion section of a samba school or a carnival street band (so called "*Bloco*" which is rhythmically comparable to percussion sections of a samba school).

1.2 Recording of stimuli

A professional musician (percussion player, composer and director of a Brazilian percussion section in Brazilian samba school) produced a composition/arrangement representing typical samba percussion sections. The composition comprises 51 bars of the first part including 4 bars of *chamada do repinique* (call of the high pitch double-headed drum), 8 bars of the first refrain (*bossa*), 59 bars of the second part and 33 bars of the second refrain. To obtain soundtracks of single instruments that could be aligned, the musician played each instrument separately while listening to a pacing sequence (metronome). For the *caixas* (snare drums) he recorded 6 different *caixas* and mixed them together in one audio track. This track was copied four times, and these four tracks were mixed/arranged again to sound like 24 *caixas* playing together. The result of this arrangement was saved in a single audio track and two of these tracks were used for the final arrangement, thus, sounding like 48 *caixas* playing in the percussion section. For the *tamborins* (small frame drums), 4 different tambourins were recorded and mixed in a single track. Two tracks were used for the final arrangement, thus, sounding like 8 *tamborins* playing in the percussion section. The instrumental part of the *repinique* (high pitch double-headed drum) consisted of two different recordings, one of the more stable part and the other for the improvising *repinique.* Three different *surdos* (low pitched bass drums - three different rhythmical versions, the first, second and third *surdo*) were recorded in three tracks. In order to have a full base sound, reverb

and equalizer effects were used. Two tracks of *agogô* (agogo bell) recordings were used (with three different rhythms that were duplicated within the track). The recorded tracks of the *chocalho (*shaker) and *cuícas* (high-pitched Brazilian friction drum) were also duplicated resulting in two tracks each for the final arrangement. Using two tracks for each instrument group allowed arrangement in space (left / right sound channel). Please note that multiple tracks of one instrument group were rendered differently by applying audio effects. The final arrangement consisted of 15 single tracks (3 tracks of *surdos* and each 2 tracks for *caixa*s, *repinique*, *cuícas*, *tambourins*, *chocalho, agogô,* see also Table 1).

Debriefing of participants revealed that they believed that the percussion section consisted of (mean ± SD) 17 ± 14 percussionists (range 5-60; median 12, percentile 25 = 6, percentile 75 = 25).

In order to obtain more descriptions of the natural recordings that can be used for a measure for variability within each instrumental part, pulse clarity and event density were computed from the recorded audio wave files using a Music Information Retrieval (MIR) toolbox 1.8.1 (Lartillot & Toiviainen, 2007[1]; Lartillot et al. 2008[2]). Pulse clarity is an estimation of the rhythmic clarity (how easily listeners can perceive the underlying rhythmic or metrical pulsation), indicating the strength of the beats based on tempo functions (autocorrelation curve computed for tempo estimation). Event density estimates the average frequency of events, i.e., the number of events detected per second. In the Table S1 below it can be seen that pulse clarity was highest for the *caixas* and lowest for the *cuíca* and *surdo* 3. Highest event density was observed for *caixas* and lowest for *surdo* 1 and 2 confirming that *surdos* 1 and 2 provide the underlying main beat at quarter notes, while *caixas* highlight pulse subdivisions by playing each 16th note with different accents (see Figure 1). The other instruments follow samba specific rhythmical patterns (see https://www.youtube.com/watch?v=Adw8u6gL2HE for a demonstration of instruments in a percussion section of Brazilian samba school).

Table S1: Pulse clarity and event density for instrumental tracks (each 151 bars).

Instrumental Track	Pulse clarity	Event density
Surdo 1	0.61	0.98
Surdo 2	0.53	0.98
Surdo 3	0.12	2.27
Caixas 1	0.87	6.99
Caixas 2	0.87	6.99
Repinique 1	0.53	3.19
Repinique 2	0.52	2.45
Cuíca 1	0.16	2.28
Cuíca 2	0.17	2.27
Tambourins 1	0.36	2.39
Tambourins 2	0.36	2.40
Chocalho 1	0.78	4.65
Chocalho 2	0.78	4.60
Agogô 1	0.38	1.67
Agogô 2	0.36	2.11

[1] Lartillot, O. & Toiviainen, P., 2007. MIR in Matlab (II): a toolbox for musical feature extraction from audio. In Proceedings of the 8th International Conference on Music Information Retrieval 237–244, 2007;

[2] Lartillot, O., Eerola, T., Toiviainen, P. & Fornari,J., 2008. Multi-feature modeling of pulse clarity: Design, validation, and optimization, International Conference on Music Information Retrieval, Philadelphia, 2008.

1.3 Control for movement during the fMRI experiment

We controlled for movements for the last five subjects, for which we had an acceleration sensor available (Brain Products GmbH[3], 3D Acceleration Sensor MR). The sensor was attached to the middle finger of the left hand and captured acceleration data (sensitivity: 420 mV/g; Supply voltage: ±5 V DC) during the fMRI scanning sessions. Root mean square (RMS) values of the *x,y,z*-acceleration values were calculated for each experimental condition (varying degrees of synchrony: 0 ms, 28 ms, 55 ms, and 83 ms delay of snare drums) and extracted for each block. A repeated measure ANOVA did not show any differences between conditions ($F(3,12) <1$). During the fMRI all participants were observed carefully from the control room through a window. No obvious movements were observed, except from one participant who was excluded from analysis also due to extensive head movements.

1.4 Stimulus presentation order in the fMRI experiment

Stimuli with the varying degrees of synchrony between instruments were presented across experimental trials in a pseudo-randomized order assuring that (1) before repeating a stimulus of the same experimental condition at least two stimuli of other experimental conditions were presented and (2) that each experimental condition is followed to an equal amount by another experimental condition (i.e., across the experiment stimuli of 83 ms asynchrony between instruments was presented 12 times and was followed each 4 times by a stimulus of 0 ms, 28 ms and 55 ms asynchrony/delay between instruments). During null events (duration 20 s), which occurred after every six trials, participants saw a fixation cross for 10 s and had no specified task and were instructed to relax.

1.5 Debriefing after fMRI scanning

After fMRI scanning, a debriefing session took place. Participants were asked which strategy they adopted to make their pleasantness ratings during scanning. Additionally, ratings for concentration, motivation, self-performance, etc. were acquired on five-point rating scales. Participants were asked whether they moved or tapped a body part with the percussion sounds during the experiment. 23 out of 24 participants indicated that they did not move or tap a body part with the percussion sounds during the experiment. One of those 23 participants reported occasional heavy breathing and another participant indicated that he sometimes had involuntary or irregular reactions/ movements, unrelated to the stimuli. One out of the 24 participants indicated that he made foot and hand movements with the stimuli throughout the entire experiment, and this participant was excluded from the sample. Furthermore, participants responded to questions about the stimuli (e.g., whether they liked the style of the recording), their musical background, their experience with percussion sections of a samba school, their emotional responses (Zentner et al. 2008) to and physiological sensations (Becker, 2004) with the typical percussion sounds of a samba school (see Methods).

1.6 fMRI parameter for anatomical images

Additionally, a set of anatomical images was acquired: (1) high-resolution T1 weighted anatomical images using a T1-weigthed three-dimensional magnetization-prepared, rapidly acquired gradient echo (MP-RAGE) sequence (voxel size 1 mm x 1 mm x 1 mm, flip angle 8°, FOV 240 mm x 240 mm x 170 mm, 170 slices, TR/TE 7.2/3.4 ms, matrix 240 x 240 sagittal slices); (2) a coronal T2 weighted anatomical image using multishot Turbo spin-echo (TSE) imaging (voxel size 0.49 mm x 0.66 mm x 4.5 mm, flip angle 90°, FOV 200 mm x 202 mm x 158 mm, 32 slices, TR: 3982 ms, TE:112 ms, matrix 408x306, coronal slices); and (3) an axial Fluid-attenuated inversion recovery (FLAIR) anatomical image using multishot Turbo spin-echo (TSE) imaging (voxel size 0.8 mm x 1.08 mm x 4.5 mm, flip angle 90°, FOV 230 mm x 182 mm x 142 mm, 26 slices, TR/TI 1100/2800 ms, TE 125 ms, matrix 288 x 168, axial slices).

[3] www.brainproducts.com

2 Supplementary Results

2.1 Behavioral experiment - evaluations

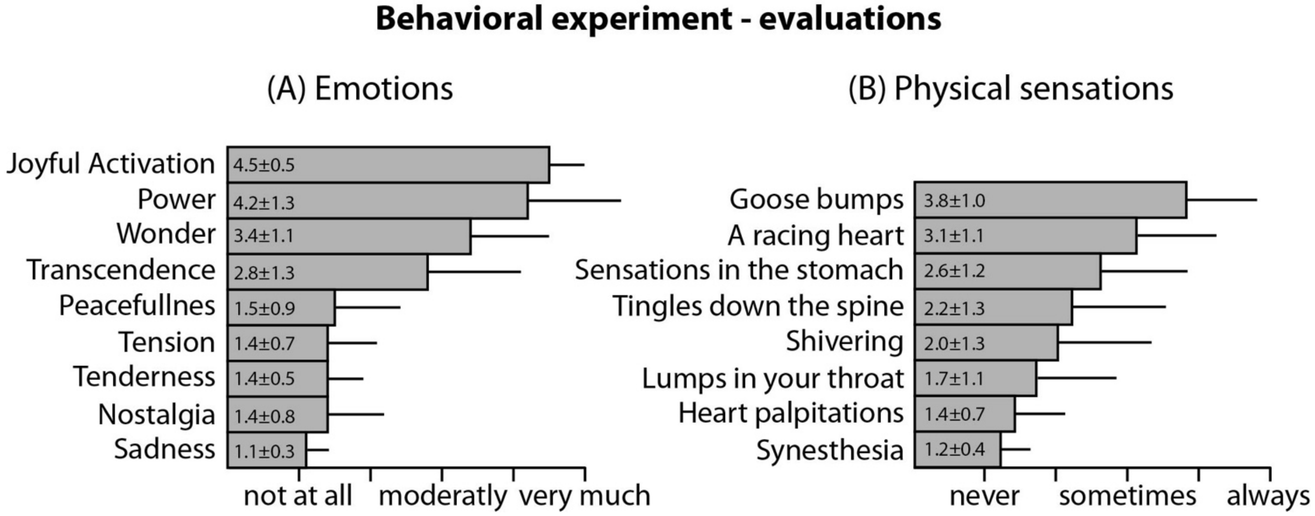

Supplementary Figure S1: Means (N=12) and standard deviations for (A) the intensity of emotions and (B) occurrence of physiological sensations normally experienced when listening to samba percussion in daily life.

2.2 fMRI experiment - evaluations

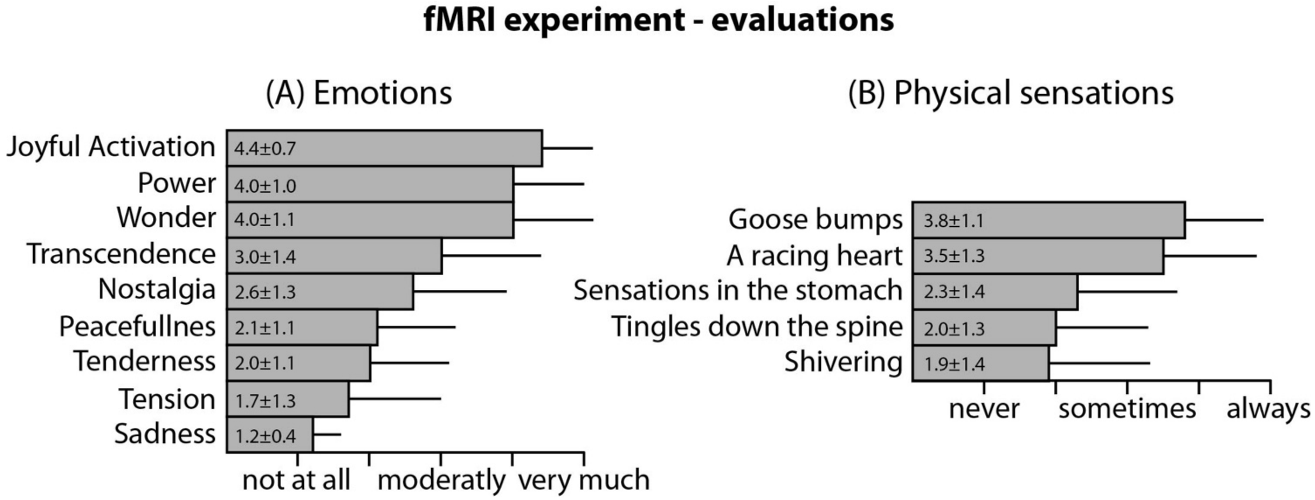

Supplementary Figure S2: Means (N=21) and standard deviations for (A) the intensity of emotions and (B) occurrence of physiological sensations normally experienced when listening to samba percussion in daily life.

2.3 Description of deep listeners and non-deep listeners by their self-reported evaluations

Those participants of the fMRI experiment (n=14) who believed that their emotional responses in general, when listening to the samba percussion in daily life are stronger than those emotional responses of most of the people they know (deep listeners), reported that samba percussion plays an important role in their life (rating on 5-point scale from 1=no role at all to 5=enormous role in life: deep listeners 3.6±1.0, non-deep listeners 2.7±0.8, t=2.1, p<.05) and that they normally feel emotions when listening to samba percussion (rating on 5-point scale from 1=not at all to 5=intensive emotions;

deep listeners 3.9±0.9, non-deep listeners 3.0±1.0, t=1.9, p<.05). Furthermore, deep listeners rated feeling more joyful activation, wonder, transcendence and nostalgia as well as greater physiological sensation of a "racing heart" (p's < .05, one-tailed testing; cf. Figure S3) when listening to samba percussion as those participants (n=7) who indicated that their emotional responses are less or equal to those emotional responses of most of the people they know (non-deep listeners).

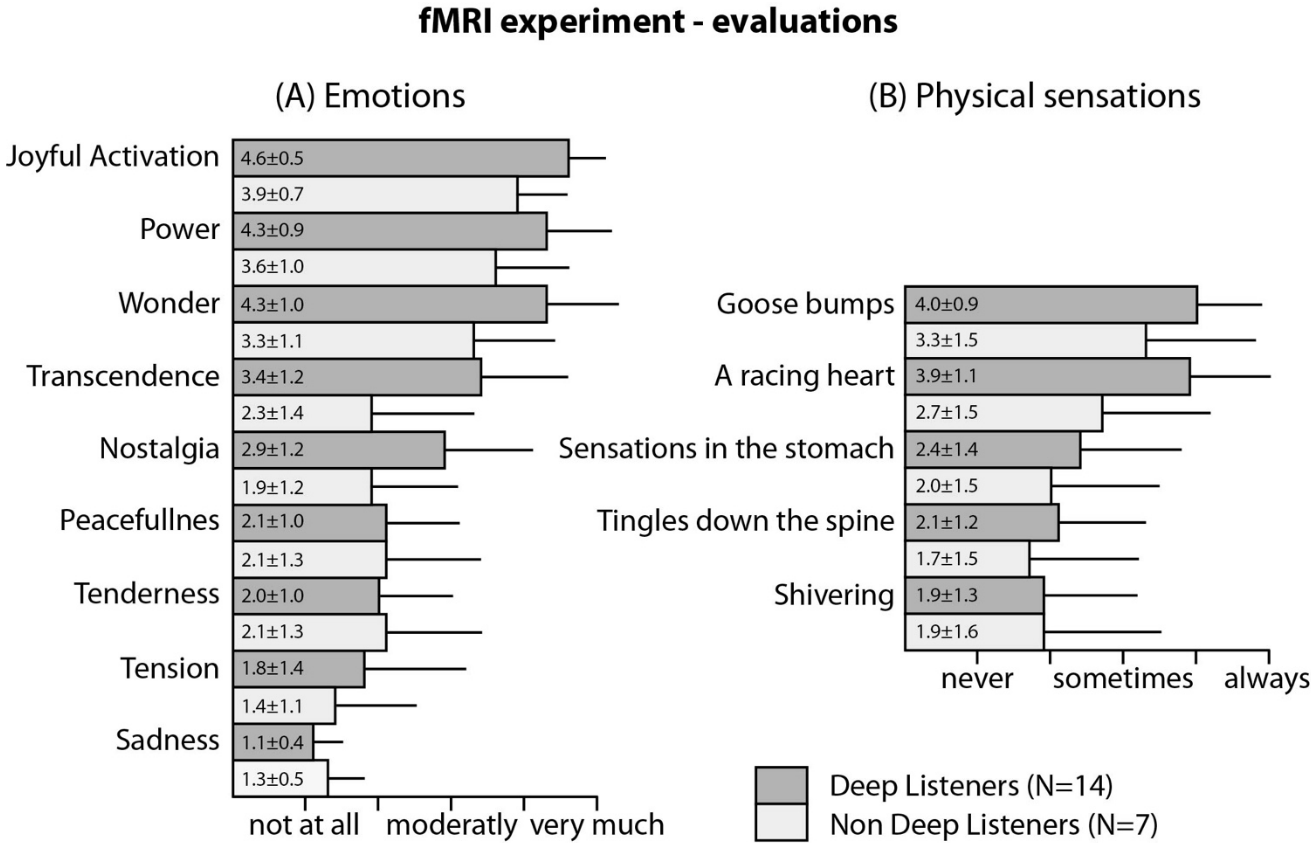

Supplementary Figure S3: Means and standard deviations for (A) the intensity of emotions and (B) occurrence of physiological sensations normally experienced in daily life when listening to samba percussion for deep listeners and non-deep listeners.

19 out of 21 participants responded to a questionnaire on entatitivity (adapted from Rusch et al. 2014). In this questionnaire participants indicated whether they perceive people who like the sounds of the percussion section of a samba school as a coherent group (e.g., "People who like the sounds of a percussion section of a samba school have many characteristics in common") and how they would consider their selves as being part of this group (e.g. "When I think about myself and my life story, I strongly consider myself a member of the group of people who like the percussion section of a samba school"). In most questions there were no differences between groups, but deep listeners indicated, that "being part of a group of people who like the sounds of the percussion section of a samba school is an important expression to whom they are" (ratings on a 9 point scale, mean ± SD: deep listeners [14] = 5.1±2.6, non-deep listeners [5 participants answered the questionnaire] = 2.2±2.7; t (17) = 2.2, p<.05).

The two groups differed also in their judgement, how much they like samba percussion in general (5 point rating scale, mean ± SD: deep listeners: 4.6±.63, non-deep listeners: 3.9 ± .69; t(19) = 2.6, p<.05), but did not differ, how often they listen to samba percussion, how familiar they are with samba percussion and its rhythm, how well they could play the rhythm, how much they pay attention when

listening to samba percussion or to which aspects they pay attention (for dancing, to know how to play, synchrony, quality, how professionally it is played, the time of rhythm). There were no further difference between the group of deep listeners and non-deep listeners in several other self-reported evaluations acquired in the debriefing for the fMRI experiment: experienced task-difficulty, motivation to make judgements during the fMRI experiment and interest in the task, concentration during listening in the fMRI experiment, thinking in other issues than the experiment, effect of listening on current emotional state, being annoyed during the fMRI experiment, how familiar the percussion excerpts were for them, the number of perceived musicians playing in the stimuli, age and education.

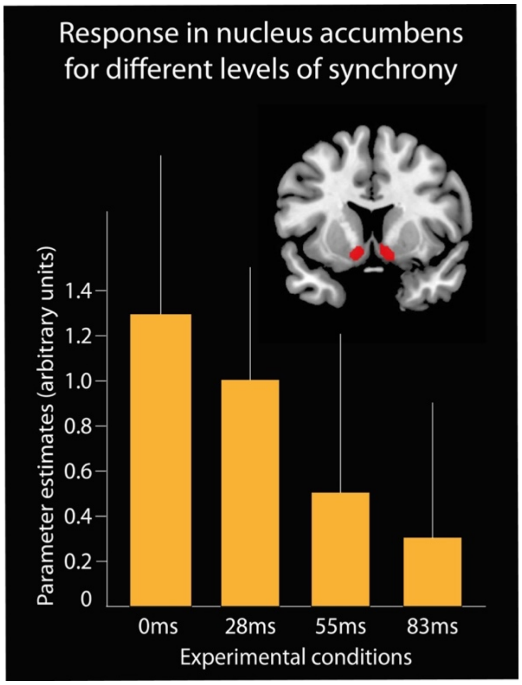

Supplementary Figure S4: Brain activation of the nucleus accumbens. Bar graphs show the mean and standard error of the mean for the parameter estimates (arbitrary units) in the nucleus accumbens (anatomical map from Pauli et al. 2018 as depicted in upper part of the figure; Values for right and left were averaged). Differences between the experimental conditions were not significant ($F_{(3,18)}$=1.59, p =.23).

Table S2: Brain areas that showed stronger activation with increasing asynchrony (contrast: 83 ms > 55 ms > 28 ms > 0 ms) at an exploratory significance level ($p<.005$ uncorrected, cluster minimum size 10 voxels).

Anatomical region	Hemi-sphere	MNI coordinates			Cluster size	*Z*-score
		x	y	z		
IFG pars orbitalis	L	-27	29	-7	*22*	*3.74*
Visual cortex	L	-12	-73	5	*76*	*3.67*
Parietal lobe	L	-30	-52	26	*15*	*3.61*
Frontal lobe (cluster reaching into IFG pars orbitalis)	R	27	26	-4	*28*	*3.47*

Note. The values shown are Montreal Neurological Institute (MNI) coordinates for activation maxima of clusters in the random effects analyses ($p < .005$, uncorrected, cluster minimum size 10 voxels). IFG, inferior frontal gyrus; R, right; L, left.

Table S3: Brain areas that showed stronger activation in the correlation analysis of better recognition of asynchronies in the test after fMRI scanning (behavioral sensitivity) and the fMRI contrast with increasing synchrony (contrast: 0 ms > 28 ms > 55 ms > 83 ms) on an exploratory significance level ($p<.005$ uncorrected, cluster minimum size 10 voxels).

Anatomical region	Hemi-sphere	MNI coordinates			Cluster size	*Z*-score
		x	y	z		
Temporal lobe/ amygdala (laterobasal part)	L	-39	-1	-25	*11*	*4.07*
Cerebellum (I-IV, V)	L	-12	-43	-19	*13*	*3.37*

Note. The values shown are Montreal Neurological Institute (MNI) coordinates for activation maxima of clusters in the random effects analyses ($p < .005$, uncorrected, cluster minimum size 10 voxels). L, left.

3 Listening Examples

Audio 1: Example of a 20-s excerpt of samba percussion with 0 ms time shift of snare drums.

Audio 2: Example of a 20-s excerpt of samba percussion with 28 ms time shift of snare drums.

Audio 3: Example of a 20-s excerpt of samba percussion with 55 ms time shift of snare drums.

Audio 4: Example of a 20-s excerpt of samba percussion with 83 ms time shift of snare drums.
